# Potential Advantages of Bioactive Compounds Extracted From Traditional Chinese Medicine to Inhibit Bone Destructions in Rheumatoid Arthritis

**DOI:** 10.3389/fphar.2020.561962

**Published:** 2020-10-07

**Authors:** Yingjie Shi, Haiyang Shu, Xinyu Wang, Hanxiao Zhao, Cheng Lu, Aiping Lu, Xiaojuan He

**Affiliations:** ^1^ Shanghai Innovation Center of TCM Health Service, Shanghai University of Traditional Chinese Medicine, Shanghai, China; ^2^ Institute of Basic Research in Clinical Medicine, China Academy of Chinese Medical Sciences, Beijing, China; ^3^ The Second Clinical Medical College, Guangzhou University of Chinese Medicine, Guangzhou, China; ^4^ College of Medicine, Southwest Jiaotong University, Chengdu, China; ^5^ School of Chinese Medicine, Law Sau Fai Institute for Advancing Translational Medicine in Bone and Joint Diseases, Hong Kong Baptist University, Hong Kong, Hong Kong

**Keywords:** ****bone destruction, rheumatoid arthritis, traditional Chinese medicine, bioactive compounds, bone metabolism

## Abstract

Bone destruction is an important pathological feature of rheumatoid arthritis (RA), which finally leads to the serious decline of life quality in RA patients. Bone metabolism imbalance is the principal factor of bone destruction in RA, which is manifested by excessive osteoclast-mediated bone resorption and inadequate osteoblast-mediated bone formation. Although current drugs alleviate the process of bone destruction to a certain extent, there are still many deficiencies. Recent studies have shown that traditional Chinese medicine (TCM) could effectively suppress bone destruction of RA. Some bioactive compounds from TCM have shown good effect on inhibiting osteoclast differentiation and promoting osteoblast proliferation. This article reviews the research progress of bioactive compounds exacted from TCM in inhibiting bone destruction of RA, so as to provide references for further clinical and scientific research.

## Introduction

Rheumatoid arthritis (RA) is a erosive autoimmune condition with lingering course (Smolen et al., 2016). Bone destruction is one of the most typical pathological features in the early RA patients, more than 10% of patients will occur pathological manifestation 8 weeks after onset (Panagopoulos and Lambrou, 2018). Current drugs for the treatment of RA are mainly non-steroidal anti-inflammatory drugs (NSAIDs), disease modifying anti-rheumatic drugs (DMARDs), hormones, biological agents and so on. Although these drugs alleviate the process of bone destruction to a certain extent, the long-term use is easy to produce side effects. Therefore, further scientific research on the treatment of bone destruction is still urgently needed. As is known to all, traditional Chinese medicine (TCM) has been used to treat RA for centuries and shown good efficacy (Guo Q. et al., 2017; Zhang Q. et al., 2019; Zhao H. et al., 2018). Recently, accumulating evidence have indicated that bioactive compounds extracted from TCM can effectively inhibit osteoclast differentiation as well as promote osteoblast proliferation, and may be used as potential therapeutically drugs. Therefore, this paper aims to review the potential therapeutic effect and targets of some representative bioactive compounds extracted from TCM, in order to provide reference for future research and development.

## Bone Metabolism Imbalance Is the Key to Bone Destruction in RA

Bone is a metabolically active organ that keeps alive through continuous renewal in the process of bone remodeling. Bone remodeling depends on the balance between bone formation and bone resorption that maintains the homeostasis of bone reconstruction (Lian, 2015). Bone destruction in RA mainly lies in excessive bone absorption and insufficient bone reconstruction. Osteoclasts play an important role in bone resorption; they are derived from monocyte/macrophage-lineage hematopoietic precursor cells which are stimulated by macrophage colony stimulating factor (M-CSF) and receptor activator of nuclear factor-kB ligand (RANKL) (Fujiwara et al., 2016). Mature osteoclasts can express proteins including integrin, artrat resistant acid phosphatase (TRAP), calcitonin receptor (CTR), cathepsin K (CTSK), and matrix metalloproteinase (MMP) at the bone surfaces that are infiltrated by synovial cells (Zhu et al., 2020a). Osteoclast differentiation is a complex multistep process, and its molecular mechanism mainly involves the regulation of inflammation mediators, transcription factors, and signal pathways. Inflammatory mediators including tumor necrosis factor-alpha (TNF-α), interleukin-1 beta (IL-1β), interleukin-6 (IL-6), interleukin-8 (IL-8), and interleukin-17 (IL-17), prostaglandin E2 (PGE2), inducible nitric oxide synthase (iNOS), etc., can promote the augment of RANKL and M-CSF after binding to the receptors on osteoclasts, so as to aggravate bone resorption in RA. Simultaneously, the initiation of osteoclast differentiation in the process of bone destruction in RA also needs the regulation of transcription factors including nuclear factor of activated T cells (NFATc1), cellular oncogene fos (c-fos), cellular oncogene jun (c-Jun) (Zhu et al., 2020a). These cytokines mediate the regulation of osteoclasts on bone destruction in RA through multiple signal pathways. For instance, TNF-α and other pro-inflammatory cytokines secreted by synoviocytes and T cells in RA promote osteoclast differentiation by activating nuclear factor kappa-B (NF-κB), mitogen-activated protein kinase (MAPK), Janus kinase/signal transducer and activator (JAK/STAT), hypoxia inducible factor-1α (HIF-1α), phosphatidylinositol-3 kinase/protein-serine-threonine kinase (PI3K/AKT), Toll-like receptor (TLR), etc. In-depth understanding of the pathological process of osteoclasts in RA, monitoring and interfering with the cytokines and signal pathways that promote osteoclast activation can provide a new target for the treatment of bone destruction in RA.

The occurrence of bone destruction in RA is not only the reason of the enhancement of bone resorption mediated by osteoclasts, but also due to the limited bone formation (bone repair) mediated by osteoblasts (Gravallese, 2017). Osteoblasts participate in osteoclasts regulation by expressing RANKL and OPG (Corrado et al., 2017). Bone marrow mesenchymal stem cells (BMSCs) are the main source of osteoblasts, BMSCs is a kind of stem cells with the potential of self-proliferation and multi-directional differentiation, which can differentiate into bone, cartilage, muscle, and other tissues under the action of different environments and stimulating factors(Komori, 2010). Because of the stimulation of cytokines such as insulin-like growth factor 1 (IGF-1) and transforming growth factor-β (TGF-β), BMSCs differentiate into osteoblast progenitor cells under the regulation of transcription factors such as Runt-related transcription factor 2 (Runx2) and bone morphogenetic protein-2 (BMP-2) (Blair et al., 2017; Komori, 2019). Subsequently, osteoblasts form osteoid, and mature osteoblasts highly express calcification-related proteins, which are mainly osteocalcin (OCN), bone morphogenetic protein-2 (BMP-2), and osteopontin (OPN) (Blair et al., 2017; Halling Linder et al., 2017; Komori, 2019). At the same time, Wnt/β-catenin, BMP/Smad, and Notch signaling pathways will act on osteoblast differentiation and maturation. Sclerostin and dickkopf-related protein 1 (Dkk-1) are the Wnt/β-catenin signaling pathway inhibitors which prevent low-density lipoprotein receptorrelated protein 5/6 (LRP 5/6) from binding to downstream signal receptor. Studies have found the expression of Wnt/β-catenin signal pathway inhibitors in osteoblasts of patients with RA was increased, which inhibited the activity of osteoblasts and promoted osteoblast apoptosis, and TNF-α and IL-1 played a promoting role (Miao et al., 2013). BMP/Smad signaling pathway can promote the formulation of osteoblasts by regulating all aspects of osteoblast cycle and has a synergistic effect with Wnt/β-catenin signaling pathway (Dejaeger et al., 2017). Miyazono et al. found that both osteoblast development and osteoblast function in BMP2/4 knockout mice were defective, and the number of osteoblasts decreased, all of which might be caused by the down-regulation of Runx2 and Osx (Miyazono et al., 2010). Verschueren et al. have proved that the total amount of phosphorylated Smad1 and Smad5 in synovium of patients with RA increased significantly compared with the control group (Verschueren et al., 2009). Many kinds of cytokines and signal pathways are interlaced with each other, and the correlation is complicated in bone metabolism. Therefore, it is the key to treat bone destruction in RA by regulating the balance between osteoclasts and osteoblasts.

## Existing Chemical and Biological Drugs for Treating Bone Destruction in RA

Once bone destruction occurs, it means that its pathological changes enter an irreversible phase, so delaying or even blocking bone destruction has become one of the main strategies for the treatment of RA. The treatment guidelines recommend the use of methotrexate (MTX) or biological disease modifying anti-rheumatic drugs (bDMARDs) first when bone destruction is found (Singh et al., 2016; Smolen et al., 2017). Conventional synthetic disease modifying anti-rheumatic drugs (csDMARDs) is regarded as the cornerstone in the treatment of RA, and it is also a first-line drug recognized by domestic and foreign guidelines (Singh et al., 2016; Smolen et al., 2017). This type of drug can effectively control the development of the disease, improve the clinical symptoms of RA and prevent the continued destruction of joint structure, but the therapeutic effect is relatively slow and it is not effective in all patients with RA (Schett et al., 2016). Glucocorticoids have strong anti-inflammatory and immuno-suppressive effects, short-acting hormones are used in the treatment of acute stage of RA (Güler-Yüksel et al., 2018). low-dose glucocorticoid can quickly relieve joint swelling and prevent joint bone destruction (Tada et al., 2016). However, unreasonable long-term use of glucocorticoids can lead to a decrease in bone mineral density and an increase in the risk of fractures (Zerbini et al., 2017; Güler-Yüksel et al., 2018). The emergence of bDMARDs can be said to be a breakthrough in the treatment of bone destruction in RA and bDMARDs have clear targeting in the treatment of bone destruction (Aletaha and Smolen, 2018). The main function of it is to antagonize the activities of T cells, B cells, osteoclasts, cytokines, and some small molecules, which delay bone destruction (Ho et al., 2019). Although the bDMARDs can correct the abnormal bone metabolism of patients to a certain extent, and improve their imaging examination and serum bone metabolism indexes, high costs reduce the dose, or frequency of bDMARDs (Burmester and Pope, 2017). In addition, they are not suitable for the complex conditions of all patients because of the single target (Zampeli et al., 2015). In summary, the current clinical application of drugs cannot adequately prevent the bone destruction in all RA patients. Due to the complexity of the bone destruction mechanism in RA, we need to explore multi-target drugs with reliable efficacy and little side effects.

## Effect of Bioactive Compounds on Bone Destruction in RA

TCM in the treatment of rheumatism has a history of thousands of years, especially the single TCM and its bioactive compounds, which is more popular in recent years, the therapeutic effect of TCM on bone destruction has gradually become a new research hotspot (Shen et al., 2019). A large number of experimental studies have been carried out, which directly or indirectly verified the role of TCM in inhibiting bone destruction in RA from different perspectives. These studies have shown that bioactive compounds extracted from TCM could down-regulate bone destruction promoting factors and up-regulate bone protective factors (Cai X. et al., 2018). Due to its diversified action ways and targets, TCM may have potential advantage in restraining RA bone destruction. Therefore, in this review, we elaborated the potential mechanisms of bioactive compounds extracted from TCM in the treatment of bone destruction in RA and divide them into alkaloids, saponins, flavonoids, and so on.

### Alkaloids

Alkaloids generally represent a highly diverse group of compounds containing cyclic structures with at least one basic nitrogen atom, which exist widely in medicinal plants, such as *Coptis chinensis Franch.*, *Sinomenium acutum (Thunb.) Rehder & E.H. Wilson*, *Conioselinum anthriscoides ‘Chuanxiong’*, *Ephedra sinica Stapf*, etc (Bednarz et al., 2019). In recent years, it has been found that alkaloids have many pharmacological activities, such as anti-inflammatory, analgesic and immunoregulation (Bach and Lee, 2019). The bone-protective alkaloids include sinomenine (SIN), tetrandrine (TET), norisoboldine (NOR), berberine, magnoflorine, ligustrazine, etc. The repair effect of SIN, TET, NOR on bone destruction in RA has been confirmed.

SIN is a kind of bioactive compound extracted from the medicinal rhizome of *Sinomenium acutum (Thunb.) Rehder & E.H. Wilson*, which has been used in the treatment of various diseases for hundreds of years. SIN is one of the strongest histamine-releasing agents, and has been proved to have anti-inflammatory, immunosuppressive, analgesic, antihypertensive, and anti-arrhythmic effects (Sun et al., 2010). In China and Japan, several SIN preparations have been used for RA in clinical practice, such as Zhengqing Fengtongning sustained-release tablets, SIN hydrochloride injection (Liu et al., 2016). The pharmacological basis of SIN in the treatment of RA lies in its anti-inflammatory, analgesic and immunosuppressive effects. Wei-Wei Liu et al. systematically evaluated the efficacy and safety of SIN in treating RA by searching the Pubmed, Cochrane Library, and other databases electronically, and including sixteen randomized controlled trials (RCTs) involving 1,500 subjects; the results of this meta-analysis indicated that SIN had better clinical efficacy and relatively fewer adverse events in the treatment of RA when compared to MTX (Liu et al., 2016). Initially, the effect of SIN on RA is the inhibition of synovitis. Related studies have confirmed that SIN exerted an effect on anti-inflammatory and immunomodulatory activities in the treatment of synovitis in RA by inhibiting pro-inflammatory cytokines, inhibiting synovial cells proliferation and T cell activation, and regulating monocyte/macrophage subsets (Zhao et al., 2007; Zhang et al., 2015; Liu et al., 2016). In addition, the preponderance of SIN in the treatment of bone destruction in RA are gradually emerging with the deepening of basic research. On one hand, SIN can indirectly inhibit bone destruction in RA by inhibiting the secretion of pro-inflammatory cytokines. on the other hand, it can directly inhibit osteoclast-mediated bone resorption (Bao et al., 2017; Liu et al., 2018). NFATc1 is one of the important transcription factors that induce osteoclast differentiation, and it can induce the formation of osteoclast specific genes such as TRAP, CTR and CTSK (Li H. et al., 2018). Recent studies have shown that NFATc1 is mainly activated through NF-κB and Ca^2+^ signaling pathways (Bendickova et al., 2017). Long-gang He et al. found that SIN inhibited the expression and transcriptional activity of NFATc1 mRNA during the differentiation of human peripheral blood mononuclear cells into osteoclasts induced by lipopolysaccharide (LPS), and the main mechanism was to inhibit the activation of NF-κB and reduce the level of Ca^2+^ in cells (He et al., 2016). In addition, SIN also reduced the breast cancer cells induced bone destruction by inhibiting the protein activity of NFATc1 (Zhang et al., 2019b). The OPG/RANKL ratio plays a decisive role in osteoclast differentiation. SIN regulated OPG/RANKL ratio induced by PGE2 and reduced the amount of TRAP-positive multinucleated osteoclasts which differentiated from RAW264.7 cells (Zhou B. et al., 2017). Another study showed that SIN obviously reduced the activation of caspase-3 and the phosphorylation of p38 (p-p38), and JNK (p-JNK) in RANKL-stimulated RAW264.7 cells, but has no effect on ERK1/2 posphorylation (He et al., 2014; Li X. et al., 2013). Xiaojuan Li et al. confirmed that SIN prevented the reduction of tissue mineral density (TMD), bone mineral density (BMD), trabecular number (Tb. N), trabecular thickness (Tb. Th), as well as the activity of TRACP5b and ALP in RA rat model induced by M. tuberculosis H37Ra (Mt) (Li X. et al., 2013). The inhibitory effect of SIN on bone resorption was also confirmed in collagen-induced arthritis (CIA) rats that SIN reduced the level of MMP-3 and MMP-13 in serum and RANKL protein expression in the synovium (Sun et al., 2014). Obviously, SIN has a good prospect and application potential in the field of clinical treatment of bone destruction in RA. However, current researches of SIN were mainly focused on its inhibitory effect on bone resorption, its effect on bone formation in RA is still unknown and needs further research. Meanwhile, the adverse effects caused by SIN through histamine release, such as allergic reactions and gastrointestinal reactions, have severely impeded the further clinical application of SIN. For people with allergic constitution, SIN should be taken in small doses and the use of SIN should be cautious; and it is suggested to avoid taking high-fat and high-protein diets during administration. For digestive tract reactions, appropriate preparation forms should be selected to alter the irritation and instability of SIN. Hence, further studies are urgently needed to explore the possibilities of decreasing the clinical adverse effects of SIN.

Besides SIN, other alkaloids also have the effect on bone destruction in RA. As a potential ligand of aryl hydrocarbon receptor (AhR), TET markedly inhibited the differentiation of RAW264.7 cells and bone marrow-derived macrophages (BMMs) into osteoclasts through AhR/c-Src/c-Cbl signal pathway. Moreover, bone mineral density (BMD) and trabecular bone (Tb) of bone parameters increased in CIA rats significantly after continuous administration of TET (Yuan et al., 2016; Jia et al., 2018; Jia Y. et al., 2019). NOR is the main isoquinoline alkaloid that inhibited the differentiation of osteoclasts *via* MAPK/NF-kB/c-fos/NFATc1, HIF, and p38/ERK/AKT/AP-1 signal pathway, and it also significantly reduced the number of TRAP-positive multinucleated osteoclasts in the joints of CIA rats as well as the levels of RANKL, IL-6, PGE2, and MMP-13 in serum of AIA rats independently of its anti-inflammatory effect, but the results also showed that NOR could not reduce the levels of OPG and MMP-1 (Luo et al., 2010; Wei et al., 2013a; Wei et al., 2013b; Wei et al., 2015). All these experimental evidences have been summarized in **Table 1**.

**Table 1:** Effects and mechanisms of alkaoids on bone destruction in rheumatoid arthritis (RA).

Bioactivecompounds	Source	Chemical structure	Targets		Functions	References
Sinomenine	*Sinomenium acutum (Thunb.) Rehder & E.H. Wilson*	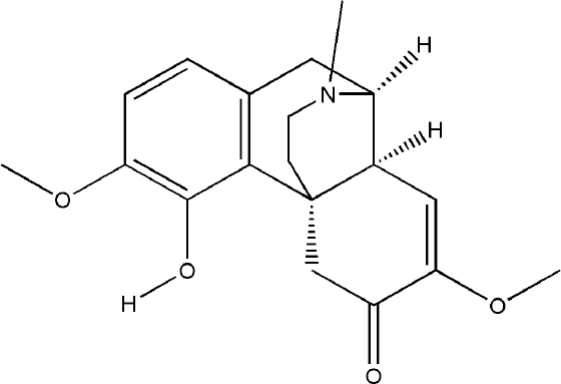		Down-regulated: GM-CSF, IL-1, IL-12, TNF-α, IL-6, RANKL, NFATc1, TRAP, MMP-9, CTSK, TLR4/TRAF6, Ca^2+^, p38MAPK-NF-κB pathway Up-regulated: OPG	inhibit osteoclast differentiation	(Liu et al., 2018) (Zhou B. et al., 2017) (Yuan et al., 2018) (He et al., 2016)
Tetrandrine	*Stephania tetrandra S. Moore*	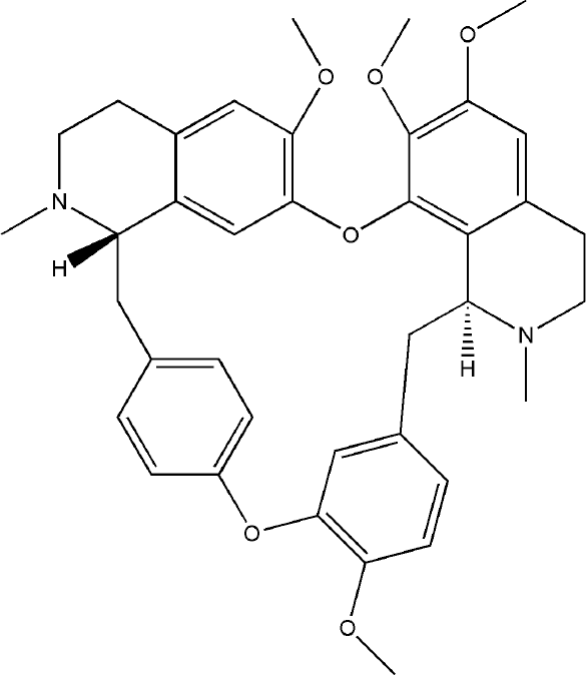		Down-regulated: NF-κB-p65, NFATc1, IFN-γ, IL-17A, Syk-PLCγ2 signaling pathway Up-regulated: IL-10, AhR nuclear translocation	inhibit osteoclast differentiation	(Jia et al., 2018) (Yuan et al., 2016) (Jia Y. et al., 2019)
Norisoboldine	*Lindera aggregata (Sims) Kosterm.*	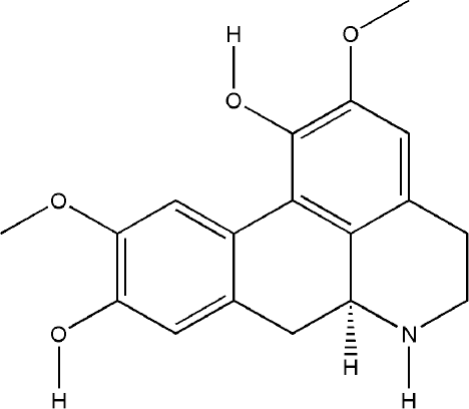		Down-regulated: RANKL, IL-6, PGE2, MMP-13, TRAF6-TAK1, p38/ERK/AKT/AP-1, MAPKs/NF-κB/c-Fos/NFATc1, HIF signal pathway	inhibit osteoclast differentiation	(Wei et al., 2013a) (Wei et al., 2013b) (Wei et al., 2015)

Berberine, magnoflorine, ligustrazine, and other alkaloids have been confirmed to inhibit the bone resorption or promote the bone formation *in vitro*, but whether they have any effect on bone destruction in RA is still unknown and needs to be verified *in vivo* (Wang et al., 2016; Wang et al., 2017; Cai Z. et al., 2018; Dinesh and Rasool, 2018). Collectively, most of the alkaloids play a role in relieving bone destruction by inhibiting the formation, differentiation and maturation of osteoclasts, and their biological activities may be related to their special structure which need to be explored by more related researches. The major challenges associated with alkaloid researches are the poor water solubility and low bioavailability which will limit their oral administration. Low bioavailability may be resolved by using semisynthetic and biochemical transformation approach. Most of the alkaloids have different biological characteristics, and biosynthesis of these agents is also varied. Therefore, it is indeed a daunting task to indicate the common mechanisms of action for alkaloids, because compounds exhibit differential cellular and molecular mechanisms even within a particular structural class. Hence, more studies *in vitro* and *in vivo* are needed to verity the effects of alkaloids agents on bone destruction in RA.

### Saponins

Saponins are linked by hydrophobic sapogenins and hydrophilic glycosyl groups through glycosides which the main components are triterpenes or spiral steranes. They have the activities of anti-inflammatory and improving body immunity (Zhao Y. et al., 2018). Saponins’ bone protection is also very prominent, triterpenoid saponins such as asperosaponin VI (ASA VI), ginsenoside Rg1, notoginsenoside R1, glycyrrhizin, and steroidal saponins such as dioscin, all of them are bone-protective saponins. The repair effect of ASA VI, ginsenoside Rg1 on bone destruction in RA has been confirmed.

ASA VI is the main bioactive compound of *Dipsacus japonicus Miq.*, which has a wide range of pharmacological effects. Its pharmacological activities in neuroprotection, prevention of osteoporosis, anti-apoptosis, analgesia, etc. that have attracted the attention of the majority of scholars, and has high research and development value (Ke et al., 2016). Liu et al. demonstrated ASA VI inhibited osteoclast differentiation to protect bone tissue, it reduced the levels of TNF-α and IL-1β in serum of CIA mice, and significantly reduced the expression of TRAP, CTSK, MMP-9 and β3-integrin involved in bone resorption, in addition, the formation of F-actin ring induced by RANKL in BMMs significantly inhibited, as well as the phosphorylation levels of AKT, JNK, and p38 (Liu et al., 2019). ASA VI not only inhibited osteoclast differentiation, but also promoted osteoblast differentiation. ASA VI induced osteoblast maturation and differentiation, and then increase bone formation in MC3T3-E1 and primary osteoblastic cells *via* increasing BMP-2 synthesis, and activating p38 and ERK1/2 (Niu et al., 2011). Ding et al. demonstrated ASA VI enhanced the ALP activity of adipose-derived stem cells (ADSCs), promoted matrix mineralization, and up-regulated the phosphorylation of bone-related proteins OCN, Runx2, and Smad2/3, which promoted the osteogenic differentiation of ADSCs (Ding et al., 2019). Although the pharmacological action of ASA VI has a wide application prospect, its development and popularization are greatly limited by its poor bioavailability. It is suggested that it can be further improved through preparation technologies such as nano-drug delivery system, sustained and controlled release drug delivery system and so on.

Ginsenoside Rg1 effectively controlled the bone damage in CIA mice, which was mainly manifested by significant decrease in the number of osteoclasts in the interphalangeal joint and ankle joint, and the expression of TRAP, CTSK, MMP and calcitonin receptor (CTR) induced by RANKL was inhibited (Gu et al., 2014). This bone-protective effect was also effective in AIA rats, after intraperitoneal injection of ginsenoside Rg1 for 14 days, the levels of TNF-α and IL-6 in the blood of AIA rats were significantly decreased. In addition, Rg1 increased the expression of peroxisome proliferators-activated receptors-gamma (PPAR-γ) protein and inhibited NF-κB nuclear translocation in RAW264.7 cells stimulated by lipopolysaccharide (LPS) (Zhang et al., 2017). All these experimental evidences have been summarized in **Table 2**.

**Table 2:** Effects and mechanisms of saponins on bone destruction in rheumatoid arthritis (RA).

Bioactivecompounds	source	Chemical structure	Targets	Functions	References
Ginsenoside Rg1	*Panax ginseng C. A. Mey*	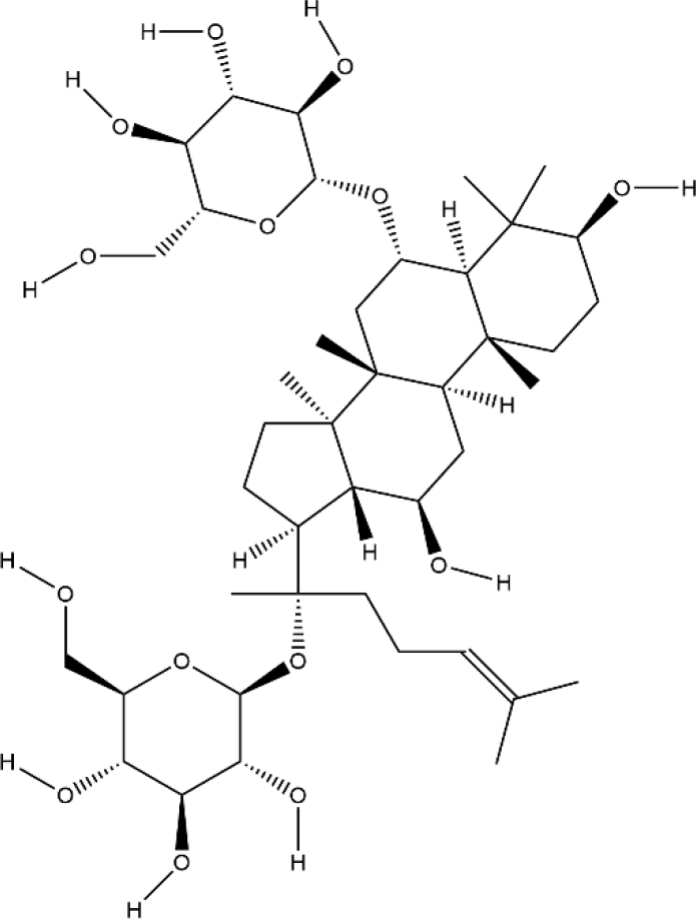	Down-regulated: NF-κB p65, MAPK, JNK, ERK1/2, P38, TNF-α, IL-6 Up-regulated: PPAR-γ	inhibit osteoclast differentiation and maturation	(Gu et al., 2014) (Zhang et al., 2017)
Asperosaponin VI	*Dipsacus japonicus Miq.*	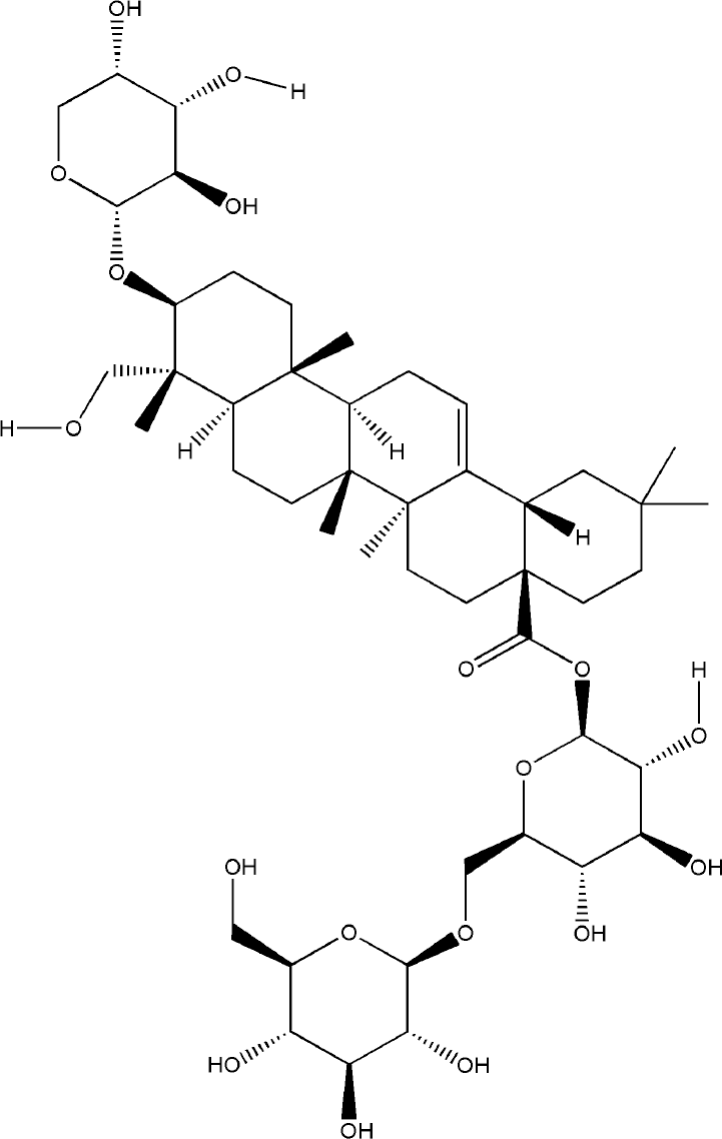	Down-regulated: NFATc1, c-Fos, TNF-α, IL-6, IL-1β, NF-κB, MAPKs, AKT pathway Up-regulated: OCN, Runx2, Smad2/3 phosphorylation	inhibit osteoclast formation; promote osteogenic differentiation	(Liu et al., 2019) (Ding et al., 2019)

*In vitro* studies, it has been proven that notoginsenoside R1, glycyrrhizin, dioscin, and other saponins can inhibit the osteoclasts’ differentiation or promote the osteoblasts’ differentiation, but their effect on bone destruction in RA requires the verification of relevant animal experiments (Li Z. et al., 2018; Qu et al., 2014; Wang et al., 2015). Based on the above findings, we can find that most of the saponins (mainly triterpenoid saponins) play a role in bone resorption or bone formation. However, it is not known whether triterpenoid saponins and steroidal saponins have different pharmacological effects on bone-protection, whether the bone protection of steroidal saponins is affected by the structural changes of liver microsomes. Therefore, a large number of related experiments are still needed. Similarly, because of the structural diversity and complexity of saponins, the acquisition of many saponins is still a difficult task, which greatly limits the further exploration of saponins’ pharmacological activity and mechanism. In addition, the bioavailability of most saponins is low after oral administration, which also brings difficulties to the research of new drugs based on active natural saponins.

### Flavonoids

Flavonoids generally refer to a series of bioactive compounds formed by the connection of two benzene rings with phenolic hydroxyl groups (A and B rings) through the central three carbon atoms. They have the effects of anti-inflammation, anti-oxidation, scavenging free radicals and so on (Wen et al., 2017). The bioactive compounds of flavonoids for protecting effect on bone by acting on osteoclasts or osteoblasts include kaempferol (KP), quercetin, icariin, poncirin, baicalin, silibinin, etc. The repair effect of KP, quercetin, icariin on bone destruction in RA has been confirmed.

KP is a natural flavonol-type flavonoid, which is present in the rhizomes of the ginger plant *Kaempferia galanga L.* According to literature reports, previous studies have shown that KP has many pharmacological effects, such as anticancer, anti-inflammatory, antioxidant, antibacterial, antiviral, immunosuppressive, etc (Calderón-Montaño et al., 2011; Jia Z. et al., 2019). KP is the basis of the quality control standard of Duanteng Yimutang preparation for clinical treatment of RA, the mechanism and molecular target of KP in the treatment of RA can provide more theoretical support and basis for its clinical application. The effect of KP on synovitis is shown that it inhibited proliferation, induced apoptosis, and ameliorated inflammation in fibroblast-like synoviocytes by suppressing the NF-κB and AKT/mTOR pathways or targeting on the fibroblast growth factor receptor 3 (FGFR3)-ribosomal S6 kinase 2 (RSK2) signaling axis (Wang J. et al., 2019). In addition to its inhibitory effect on synovitis, KP also showed bone-protecting effect in the treatment of RA. KP’s bone-protective function in RA is to inhibit pro-inflammatory cytokines indirectly, it also can directly act on osteoclast-mediated bone resorption or osteoblast-mediated bone formation. Alice Wattel et al. explored the effect of KP on bone resorption for the first time, they found KP directly induced apoptosis of mature osteoclasts in the highly purified rabbit osteoclasts, and its estrogenic effect could be involved in the inhibition of bone resorption (Wattel et al., 2003). KP inhibited RANKL-induced expression of c-fos, c-RANK and CTR in RAW264.7 cells, however, TNF-α-stimulated intracellular ROS production was unaltered by KP (Pang et al., 2006). KP inhibited IL-1β-stimulated, RANKL-mediated the expression of NFATc1, phosphorylation of ERK 1/2, p38 and JNK MAP kinases in bone marrow cells (Lee et al., 2014). Autophagy has pivotal roles in maintaining bone metabolic balance. Sequestosome 1 (p62/SQSTM1) is an important bridge protein that becomes incorporated into autophagosomes in RANKL-induced autophagy and osteoclastogenesis (Rea et al., 2013). Kim et al. found KP inhibited autophagy and promoted apoptotic cell death in RAW 264.7 cells by the degradation of p62/SQSTM1. KP’s main manifestation of inhibiting osteoclastogenesis was to abrogate the formation of TRAP-positive multinucleated cells induced by RANKL in this *in vitro* experiment (Kim C. J. et al., 2018). There are studies that showed the direct effects of KP on osteoblastic cells or osteoblastic precursor cells by different mechanisms. KP’s estrogenic effect acted on osteoblast differentiation, KP induced the activity of osteoblast differentiation biomarkers including ALP, OCN, osterix, Runx2 by estrogen receptor activation in rat primary osteoblasts (Guo et al., 2012). Interestingly, KP-mediated autophagy promotes osteoblast differentiation and bone mineralization. Kim et al. found KP increased the expression of the autophagy-related factors beclin-1, p62/SQSTM1, and the expression of osteoblast-related factors Runx2, osterix, BMP-2, and collagen I also decreased with dose dependent under the concentration of 10 μM in MC3T3-E1 cells (Kim et al., 2016). With further insights into the mechanism of bone-protective action of KP, Yang Wang et al. found KP’s regulation of Wnt/β-catenin pathway was to up-regulate the microRNA-101 in MC3T3-E1 cells (Wang Y. et al., 2019). The effect of KP on bone destruction in RA has also been confirmed *in vivo*. After intragastric administration of KP, the effect of synovitis on the invasion of surrounding bone and the level of MMP were suppressed in CIA model (Pan et al., 2018). Furthermore, KP inhibited the progressive structural destruction of RA joints by blocking the bFGF/FGFR3/RSK2 signaling axis in CIA model, the mainly manifest was shown as decreased the levels of osteoclast specific genes TRAP, CTR, CTSK, c-jun, and p50 (Lee et al., 2018). Though KP’s poor bioavailability represents a significant obstacle, the use of KP-based nanoparticles has brought more hope on chemoprevention strategies. While KP shows potential for improving bone destruction by the alterations of osteoclast or osteoblast related protein genes or RNAs, but most of the research conducted on KP resistance to bone destruction potency was *in vitro*, making it difficult to draw a final conclusion on its usefulness, *in vivo* studies and clinical trials are scarce so far, thus stressing the need for more in-depth experiments.

Quercetin and icariin are two other flavonoids with the effect of treating bone destruction in RA. Quercetin not only inhibited the expression of osteoclast-specific genes TRAP, CTSK, NFATc1 *in vitro*, and the plasma level of MMP-3, MMP-9 in CIA mice, but also up-regulated the mRNA and protein expression of osteoblast-specific genes Osx, Runx2, ALP and OCN (Guo C. et al., 2017; Haleagrahara et al., 2018; Kim H. R. et al., 2019). Icariin blocked osteoclast generation by inhibiting the expression of TRAF6 in the early stage of osteoclast formation and the activation of ERK1/2 and NF-κB. In addition, the decrease of F-actin ring formation revealed that bone resorption capacity of mature osteoclasts was inhibited by Icariin. Moreover, Icariin’s inhibitory effect on bone resorption in RA has also been confirmed in CIA model (Chi et al., 2014; Kim B. et al., 2018; Xu et al., 2019). All these experimental evidences have been summarized in **Table 3**.

**Table 3:** Effects and mechanisms of flavonoids on bone destruction in rheumatoid arthritis (RA).

Bioactivecompounds	Source	Chemical structure	Targets	Functions	References
Kaempferol	*Kaempferia galanga L.*	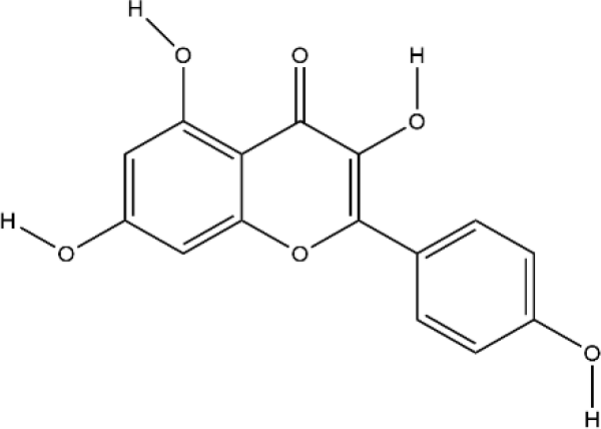	Down-regulated: MMP-1, MMP-3, COX-2, PGE2, ERK-1/2, p38, JNK, NF-κB, TRAP, CTR, MAPKs, c-Fos, NFATc1, CTSK, c-Jun Up-regulated: collagen I, Runx2, Osx, BMP-2, ATG5, beclin-1, LC3	inhibit osteoclast differentiation; promote osteoblast differentiation	(Yoon et al., 2013) (Lee et al., 2018) (Lee et al., 2014) (Kim et al., 2016)
Quercetin	*Sophora japonica L.*	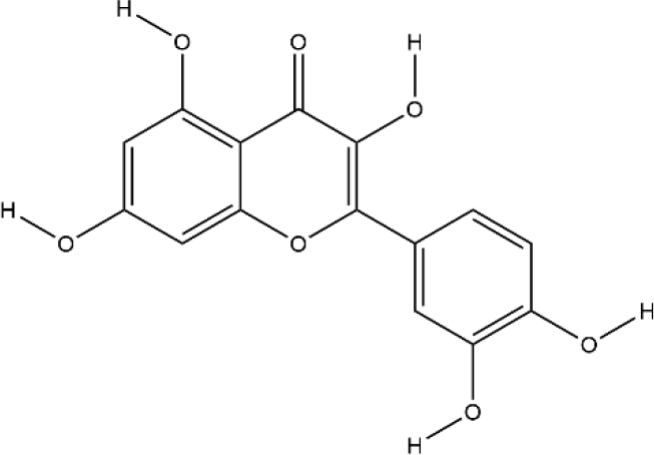	Down-regulated: TNF-α, IL-1β, MCP-1, IL-17, ERK, IκBα, TRAP, CTSK, DC-STAMP, NFATc1, OC-STAMP, caspase3 Up-regulated: Wnt/β-catenin signaling pathway	inhibit osteoclast differentiation; promote osteoblast differentiation and inhibit osteoblast apoptosis	(Kim et al., 2019) (Guo C. et al., 2017)
Icariin	*Epimedium brevicornu Maxim.*	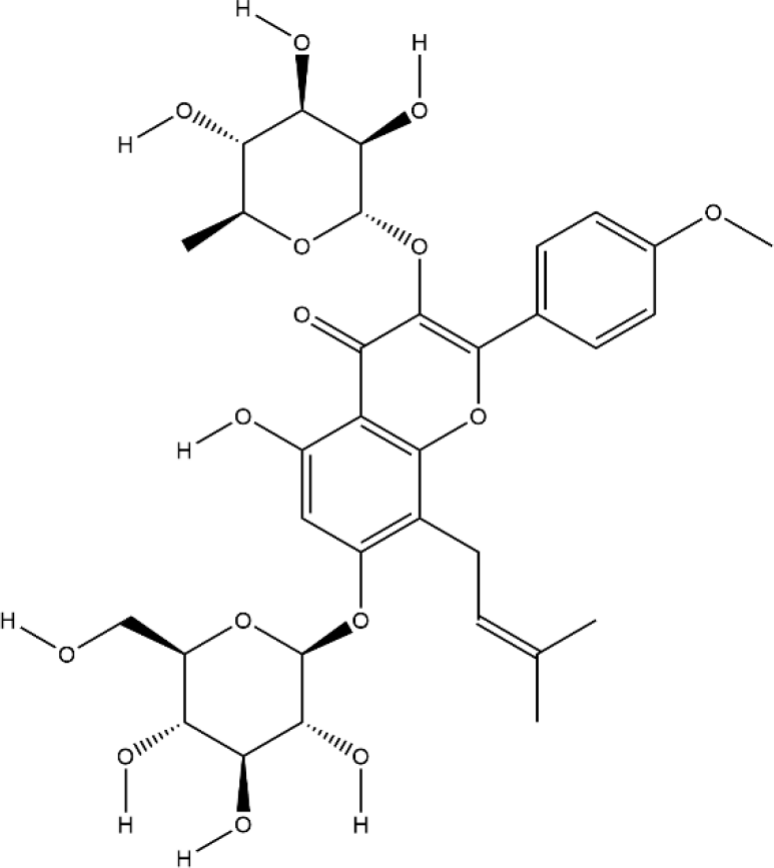	Down-regulated: TRAF6, ERK phosphorylation, NF-κB, MAPK signaling pathway	inhibit osteoclast formation and differentiation	(Kim B. et al., 2018) (Xu et al., 2019) (Hsieh et al., 2011)

Poncirin, baicalin, silibinin, and other flavonoids have been shown significant effects on osteoclasts or osteoblasts (Kim et al., 2009; Lu et al., 2017; Chun et al., 2020). But whether they can repair bone destruction in RA is not verified which need a large number of relevant animal experiments. Actually, most of the flavonoids play a role in bone resorption or bone formation by different signal transduction mechanisms. Autophagy may be involved in, but still need conduct appropriate animal experiments. The difference between protective autophagy and inhibitory autophagy induced by flavonoids may be related to the type, mode and dose of flavonoids, as well as the type and the state of the cell lines. Moreover, most flavonoids have low cytotoxicity to normal cells at normal dose, and are safer than traditional cytotoxic drugs, so they have strong potential for clinical application.

### Terpenoids

Terpenoids, a kind of compounds with isoprene unit (C5 unit) as the basic structural unit in the molecular framework, have anti-inflammatory, immunoregulatory and other pharmacological activities (Guesmi et al., 2017). The bioactive compounds of terpenoids for protecting effect on bone include triptolide (TP), celastrol, artesunate, parthenolide, andrographolide (AP), etc. The repair effect of TP, celastrol, artesunate on bone destruction in RA has been confirmed.

TP (a dierpene triepoxide in chemical structure), extracted from *Tripterygium wilfordii Hook.f.*, is a kind of natural product with various biological activities. It attracted worldwide attention in the 1960s because of its pharmacological effects in a variety of diseases such as RA, no small cell lung cancer, and refractory nephrotic syndrome (Yuan et al., 2019). TP has been considered as a promising anti-RA drug which has definite effects including immunosuppression, anti-inflammatory reaction, inducing apoptosis, inhibiting angiogenesis (Li et al., 2014). Tripterygium wilfordii tablets, Tripterygium wilfordii glycosides tablets, and Tripterygium hypoglaucum hutch tablets which take triptolide as the quality control standard are available in clinic (Law et al., 2011). The pharmacological effect of TP on synovitis is the focus of researchers to explore the mechanism of triptolide in the treatment of RA at the very start. TP treated synovitis in RA by regulating immune-related cells (such as T cells, macrophages, dendritic cells), immune-related inflammatory mediators and immune-related angiogenesis (Chan et al., 1999; Zhu et al., 2005; Kong et al., 2013). With the research developed, researchers found that delaying or even blocking bone destruction is another primary mechanism of TP in the treatment of RA. Zhu et al. systematically evaluated the effect of Tripterygium wilfordii glycosides tablets in the treatment of RA by searching the Pubmed, Web of Science, Cochrane Library and other databases, three RCTs were employed which involved a total of 223 subjects, and the results indicated that Tripterygium wilfordii glycosides tablets had a good effect on regulating the modified Sharp score (m TSS), tender join joint erosions (JE) and joint space narrowing (JSN), and the effect is better than the positive drugs MTX and sulfasalazine, which reflected the advantages of TP in the treatment of bone destruction in RA (Zhu G. Z. et al., 2019). As a typical anti-inflammatory drug, TP indirectly treated bone destruction in RA by inhibiting the levels of pro-inflammatory cytokines such as TNF-α and IL-1β and promoting the secretion of IL-10 and TGF-β1 derived from T cells (Xu et al., 2016). RANK-RANKL signaling activates a variety of downstream signaling pathways required for osteoclast development. TP suppressed RANKL-induced NF-κB activation in osteoclast precursor cells by inhibiting IκBα kinase activation, IκBα phosphorylation, and IκBα degradation effectively, and osteoclast formation induced by tumor cells was inhibited (Park, 2014). Spleen cells are also one of the main sources of osteoclast precursors, low-dose TP promoted the apoptosis of osteoclast precursors by inhibiting the overexpression of cellular inhibitor of apoptosis protein 2 (cIAP2) in fresh spleen cells induced by M-CSF (Wang et al., 2018). AKT-MDM2‐induced cell death might contribute to the osteoclastogenesis suppression. Cui et al. found TP suppressed NFATc1 overexpression and AKT phosphorylation when PI3K-AKT-NFATc1 pathway was activated induced by RANKL in BMMCs or RAW264.7 cells (Cui et al., 2020). The therapeutic effect of TP on bone destruction in RA has been confirmed *in vivo*, TP improved bone destruction of TNF-Tg mice by decreasing the levels of pro-inﬂammatory cytokines, promoting the apoptosis of osteoclast precursors and inhibiting the generation of osteoclast (Wang et al., 2018). The result of Micro CT showed that TP significantly increased joint bone density, bone volume fraction and trabecular thickness of CIA mice, reduced trabecular separation of inflammatory joints through inhibiting the expression of RANKL and increasing the expression of OPG (Liu et al., 2013). Although TP has already been proved to have potential advantages in the treatment of bone destruction in RA *in vitro* and *in vivo*, its precise molecular targets that responsible for the potent biological activity have not been fully identified yet. At the same time, the side effects of TP are to block its clinical application to a great extent, development of efficient TP-targeted delivery system is an available strategy to realize targeted delivery of TP with reduced toxicity.

Celastrol and artesunate are two other terpenoids with the effect of treating bone destruction in RA. Celastrol played an inhibitory effect against the formation and function of osteoclasts by regulating the ratio of RANKL/OPG and the expression of transcription factors in osteoclasts induced by RANKL, the main mechanisms involved the phosphorylation of NF-κB and MAPK (Nanjundaiah et al., 2012; Gan et al., 2015; Cascao et al., 2017). Artesunate down-regulated the expression of osteoclast-specific genes TRAP, CTSK, c-fos, and NFATc, as well as the expression of MMP-9 protein in CIA model hind paw by inhibiting the ERK and JNK phosphorylation (Li Y. et al., 2013; Wei et al., 2018). All these experimental evidences have been summarized in **Table 4**.

**Table 4:** Effects and mechanisms of terpenoids on bone destruction in rheumatoid arthritis (RA).

Bioactivecompounds	Source	Chemical structure	Targets	Functions	References
Triptolide	*Tripterygium wilfordii Hook.f.*	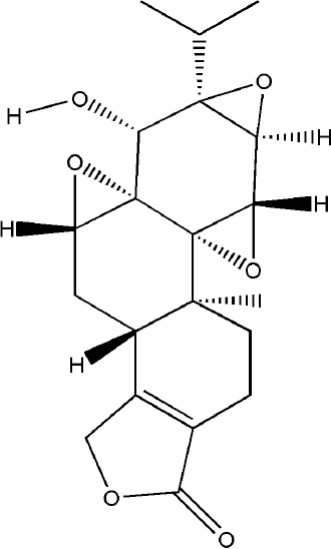	Down-regulated: IL-1α, IL-1β, TNF-α, cIAP2, RANKL Up-regulated: IL-10, TGF-β1, OPG	promote the apoptosis of osteoclast and inhibit osteoclast differentiation	(Wang et al., 2018) (Wang S. et al., 2019) (Liu et al., 2013) (Xu et al., 2016)
Celastrol	*Tripterygium wilfordii Hook.f.*	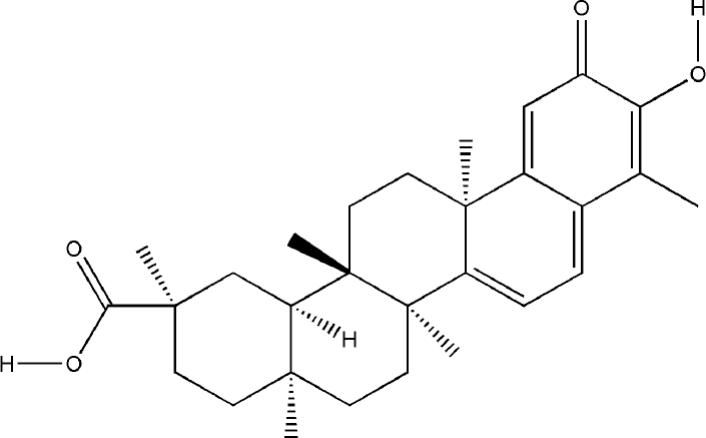	Down-regulated: IL-6, IL-1β, NF-κB, 90β protein, c-Fos, c-Jun, NFATc1, TRAP, CTSK, CTR, MMP-9, RANKL, GM-CSF, M-CSF, OPN, IGF-1, MMP-9	inhibit osteoclast differentiation and function	(Astry et al., 2015) (Cascao et al., 2017) (Gan et al., 2015) (Nanjundaiah et al., 2012)
Artesunate	*Artemisia annua L.*	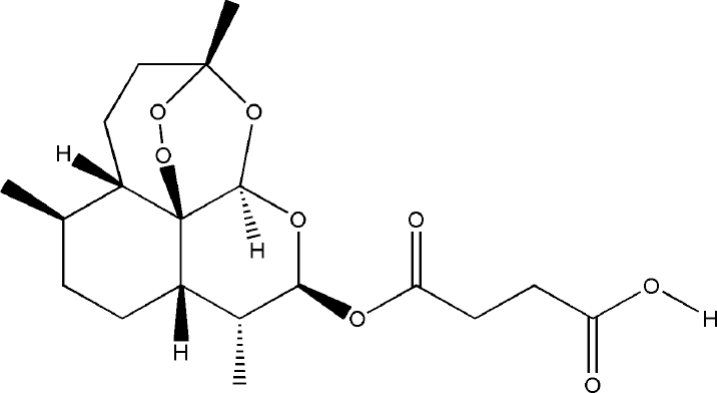	Down-regulated: MMP-9, TNF-α, IL-1β, IL-17, ERK, JNK, TRAP, CTSK, c-Fos, NFATc1	inhibit osteoclast differentiation	(Li Y. et al., 2013) (Wei et al., 2018)

Parthenolide, AP and other terpenoids exerting an effect on osteoclasts or osteoblasts has been found *in vitro* (Kim et al., 2014; Qu et al., 2014; Zhang et al., 2014; Li et al., 2016). But whether they have any effect on bone destruction in RA is unknown, it is critical to verified terpenoids’ pharmacological action of bone protection *in vivo*. Terpenoids are expected to become the main drugs for the treatment of bone destruction in RA with its significant pharmacological effects, low toxicity and side effects, but the efforts to further improve terpenoids’ efficacy are limited because of the unclear structure-activity relationship. Therefore, it is necessary to explore new technical measures to definite the structure-activity relationship.

### Phenols

Phenols is a kind of bioactive compound, and its hydroxyl group is directly connected to benzene ring or other aromatic ring. Phenols has strong effects of anti-oxidation, anti-atherosclerosis, anti-infection, anti-tumor, and anti-osteoporosis (Zenkov et al., 2016). The bioactive compounds of terpenoids for protecting effect on bone by acting on osteoclasts or osteoblasts including resveratrol (RES), ferulic acid (FA), curcumin, gastrodin, paeonol, etc. The repair effect of RES, FA on bone destruction in RA has been confirmed.

RES, extracted from *Reynoutria japonica Houtt.*, is a naturally occurring polyphenolic compound containing stilbene structure. It has reported that RES has positive effects on health and increase life span (Baur and Sinclair, 2006). RES mainly functioned on the centrum restraint, heart sturdiness, inflammation diminishing, and anti-cancer (Ko et al., 2017; Wahab et al., 2017). At present, the role of RES in the treatment of RA is particularly remarkable because of its unique anti-inflammatory and immunosuppressive pharmacological effects. RES treated RA by enhancing the apoptosis of fibroblast-like synoviocytes, inhibiting angiogenesis, etc, the mechanism of its inhibition of synovitis included the regulation of NF-κB, MAPK-p38, JAK/STAT, PI3K/AKT, etc signaling pathways (Yang et al., 2017; Yang et al., 2018; Zhang et al., 2019a). As a natural phytoestrogen, RES acts as an estrogen receptor agonist which obviously promotes bone growth under normal bone growth environment and protects bone under weightlessness and diseases (Baur and Sinclair, 2006). The dosage forms of RES used for RCT to investigate the effects of RES on bone in type 2 diabetic patients or metabolic syndrome (MetS) include RES tablets and RES capsules, these clinical trials further confirmed the protective effect of RES on bone (Ornstrup et al., 2014; Bo et al., 2018). RES is reported to impact bone destruction by increasing osteoblast differentiation and function *in vitro*. RES at non-toxic concentrations dose-dependently inhibited RANKL-induced osteoclast differentiation and induced osteoclast apoptosis by inhibition of ROS generation (He et al., 2010). RES improved the oxidative stress state of RAW264.7 cells, thus inhibited the mRNA expression of osteoclast specific enzyme MMP-9, TRAP, CTSK, this was the first time to confirm that RES promoted resistance to oxidative damage and restrained osteoclastogenesis by inhibiting the PI3K/AKT signaling pathway at the molecular level (Feng et al., 2018). The role of RES in promoting osteoblast differentiation may be more prominent. RES suppressed OCN synthesis in osteoblasts induced by stimulating factors (triiodothyronine or BMP-4) *via* the activation of SIRT1 or the amplification of p38 MAP kinase activity (Kuroyanagi et al., 2015; Fujita et al., 2017). Although RES indirectly promoted osteoblast differentiation by inhibiting inflammation, RES promoted the increase of ALP and OPG in BMSCs induced by LPS, but did not decrease the levels of IL-6 and IL-8, the result indicated RES’s effect on osteoblasts could be independent of inflammation. Meanwhile, the Wnt/β-catenin and ERK/MAPK signaling pathways also participated in the mechanism of RES’s bone-protection (Ornstrup et al., 2016; Zhao X. E. et al., 2018). Silent information regulator 2 homologue 1 (SIRT1) is a positive regulator of the master osteoblast transcription factor, RES reduced the decrease of OCN, OPN, and RUNX2 expression in MC3T3-E1 cells induced by LPS, and the main possible mechanism was to regulate mitochondrial function of osteoblasts by increasing the expression of SIRT1 (Ma et al., 2018). Yaqiong Yu et al. found a new mechanism of RES promoting osteoblast differentiation under the same result, the activation of AMP-activated protein kinase (AMPK) phosphorylation and inhibitor of suppressor of cytokine signaling 1 (SOCS1) were important signal events that RES inhibited LPS-induced MMP-2 production in MC3T3-E1 cells (Yu et al., 2018). The potential protective effects of RES on bone destruction in RA has been confirmed *in vivo*, RES significantly improved the narrowing of joint space, and the expression level of MMP1 and MMP13 in the synovial tissue was significantly reduced in CIA rats (Hao et al., 2017). Similarly, the expressions of MAPK, Src kinase, STAT3, and Wnt5a in the CIA model joint tissue also participated in the repairing effect of RES on bone destruction in RA (Oz et al., 2019). More and more experimental studies have emphasized the immunomodulatory and osteoprotective effects of RES *in vivo* and *in vitro*. Although these studies have produced exciting results, we still faced with some problems such as poor water solubility and low bioavailability. Therefore, various strategies are being implemented, including the development of RES-related preparations (nanoparticles, liposomes, micelles and phospholipid complexes, etc.) to improve their bioavailability. In addition, several other methods have been used to improve its bioavailability, including changing the route of administration of resveratrol and blocking the metabolic pathway through treatment with other drugs. In fact, since RES has multiple intracellular targets, additional data are needed to determine the results of interactions or synergies between other polyphenols.

FA has the functions of anti-inflammatory, anti-oxidation, inhibiting platelet aggregation, improving microcirculation, and so on (Zheng et al., 2019; Perez-Ternero et al., 2017). Zhu et al. found FA significantly alleviated joint swelling and reversed the increase of C-reactive protein (CRP) and rheumatoid factor (RF) in CFA rats, its protective mechanism on joints is mainly to reduce the secretion of TNF-α and increase the secretion of TGF-β by inhibiting JAK/STAT pathway (Zhu et al., 2020b). Sagar et al. found that FA inhibited the expression of DC-STAMP which is necessary for the differentiation and maturation of osteoclasts, as well as inhibited RANKL-induced upregulation of MMP-9 and CTSK. In addition, it induced mature osteoclast apoptosis through the caspase-3 pathway (Sagar et al., 2016). Scanning electron microscopy and TRAP staining analysis showed that FA significantly inhibited the osteoclast differentiation induced by RANKL, it inhibited the formation of mature osteoclasts by inhibiting the expression of NFATc1 and c-fos, it further inhibited the bone resorption activity of mature osteoclasts by inhibiting the expression of TRAP, MMP-9, and CTSK (Doss et al., 2018). All these experimental evidences have been summarized in **Table 5**.

**Table 5:** Effects and mechanisms of phenols on bone destruction in rheumatoid arthritis (RA).

Bioactivecompounds	Source	Chemical structure	Targets	Functions	References
Resveratrol	*Reynoutria japonica Houtt.*	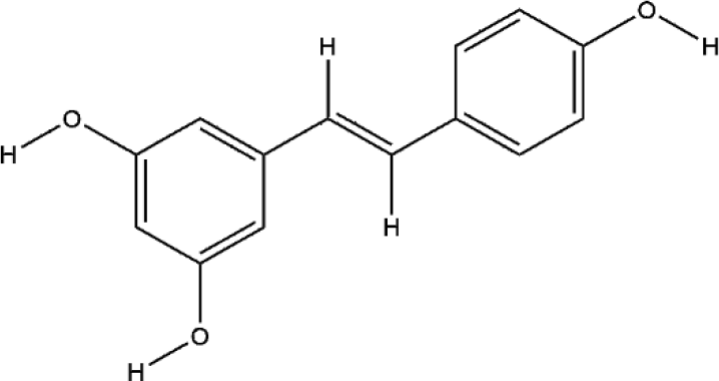	Down-regulated: ROS, MMP-9, TRAP, CTSK, PI3K/AKT, MAPK signaling pathway Up-regulated: ALP, OPG, OCN, OPN, RUNX2, Wnt/β-catenin signaling pathway	inhibit osteoclast differentiation; promote osteoblast differentiation	(He et al., 2010) (Feng et al., 2018) (Zhao X. E. et al., 2018). (Ma et al., 2018). (Hao et al., 2017)
Ferulic acid	*Ferula assa-foetida L.*	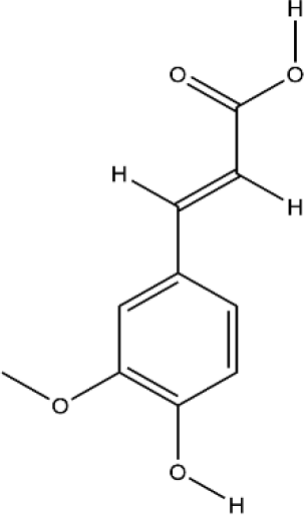	Down-regulated: TNF-α, JAK2, MMP-9, CTSK, NFATc1, c-Fos, TRAP, NF-κB signaling pathway Up-regulated: TGF-β, caspase-3	inhibit osteoclast differentiation and mature	(Zhu L. et al., 2019) (Sagar et al., 2016) (Doss et al., 2018)

*In vitro* studies, it has been found that curcumin, gastrodin, paeonol and other phenols can also inhibit bone resorption or promote bone formation (Zhou F. et al., 2017; Li et al., 2019; Wang Q. et al., 2019). However, we need more experiments *in vivo* to explore their effects on bone destruction in RA. Phenols has good antioxidant activity because of the high reactivity of hydroxyl substitution and the ability to engulf free radicals. It is known that oxidative stress can improve the activity of osteoclasts, whether phenols’ antioxidant properties are closely related to its pharmacological effects on osteoclasts or osteoblasts needs to be further studied.

## Conclusion and Further Perspectives

Bone destruction in RA is difficult to cure, and the disability rate is high, which is a serious threat to human health. Therefore, it is particularly important to find more effective and reliable treatment methods and means. TCM in the treatment of RA has a long history, the research of TCM in the treatment of bone destruction in RA has made rapid progress, which shows that TCM has strong advantages and characteristics in the treatment of bone destruction in RA. These bioactive compounds extracted from TCM display anti-bone destructive activity *in vitro* and *in vivo*, and they have shown very good results from different aspects. The potential of bioactive compounds extracted from TCM to provide or inspire the development of anti-bone destruction bioactive drugs is, therefore, really quite evident. However, the biological tests about these compounds and test results are different, mainly due to the different extraction protocols, compounds purity and intervention projects (including doses, animal, or cell models, test methods, and so on). Even compounds with the same purity in the study may have different test results and will make people doubt the authenticity of these tests. Therefore, it has become necessary to use some advanced and interdisciplinary technology and methodology unify extraction protocols and purity identification standard. Furthermore, the studies on compounds are only in the early stage, most of them are focused on *in vitro* experiments. Hence, additional investigation into pharmacokinetics with animal models and clinical studies are necessary. In addition, proper dosage needs to be considered to prevent the potential toxicity when developing these compounds into clinically viable drugs. Similar compounds can treat bone destruction of RA through different signal transduction mechanisms. Whether these mechanisms are interrelated, and whether compounds with the same or similar structures have similar pharmacological effects on osteoclasts or osteoblasts remain to be further verified. Combinations of different compounds that regulate bone destruction in RA through different mechanisms may have synergistic or cumulative effects. This also needs to be further verified.

It is hoped that this review can highlight the importance of bioactive compounds extracted from TCM in the treatment of bone destruction in RA and provide a new direction for future researchers. In the future, we need advanced technology to separate more bioactive compounds from TCM for the treatment of bone destruction in RA, and further explore the exact molecular mechanism and therapeutic targets of the bioactive compounds, which will be helpful for the treatment of bone destruction in the early stage, preventing disability and enhancing the quality of patients’ life.

## Author Contributions

YS wrote the manuscript. HS, XW, and HZ contributed to the literature research for the manuscript. CL revised the manuscript. AL and XH revised and approved the manuscript. All authors contributed to the article and approved the submitted version.

## Funding

This work was supported by the National Key R&D Program of China (2018YFC1705205).

## Conflict of Interest

The authors declare that the research was conducted in the absence of any commercial or financial relationships that could be construed as a potential conflict of interest.

## References

[B1] Aletaha D. Smolen J. S. (2018). Diagnosis and Management of Rheumatoid Arthritis: A Review. Jama 320, 1360–1372. 10.1001/jama.2018.13103 30285183

[B2] Astry B. Venkatesha S. H. Laurence A. Christensen-Quick A. Garzino-Demo A. Frieman M. B. (2015). Celastrol, a Chinese herbal compound, controls autoimmune inflammation by altering the balance of pathogenic and regulatory T cells in the target organ. Clin. Immunol. 157, 228–238. 10.1016/j.clim.2015.01.011 25660987 PMC4410084

[B3] Bach D. H. Lee S. K. (2019). The Potential Impacts of Tylophora Alkaloids and their Derivatives in Modulating Inflammation, Viral Infections, and Cancer. Curr. Med. Chem. 26, 4709–4725. 10.2174/0929867325666180726123339 30047325

[B4] Bao B. H. Kang A. Zhao Y. Shen Q. Li J. S. Di L. Q. (2017). A selective HPLC-MS/MS method for quantification of SND-117 in rat plasma and its application to a pharmacokinetic study. J. Chromatogr. B. Analyt. Technol. Biomed. Life Sci. 1052, 60–65. 10.1016/j.jchromb.2017.03.008 28359984

[B5] Baur J. A. Sinclair D. A. (2006). Therapeutic potential of resveratrol: the in vivo evidence. Nat. Rev. Drug Discovery 5, 493–506. 10.1038/nrd2060 16732220

[B6] Bednarz H. Roloff N. Niehaus K. (2019). Mass Spectrometry Imaging of the Spatial and Temporal Localization of Alkaloids in Nightshades. J. Agric. Food Chem. 67, 13470–13477. 10.1021/acs.jafc.9b01155 31334645

[B7] Bendickova K. Tidu F. Fric J. (2017). Calcineurin-NFAT signalling in myeloid leucocytes: new prospects and pitfalls in immunosuppressive therapy. EMBO Mol. Med. 9, 990–999. 10.15252/emmm.201707698 28606994 PMC5538425

[B8] Blair H. C. Larrouture Q. C. Li Y. Lin H. Beer-Stoltz D. Liu L. (2017). Osteoblast Differentiation and Bone Matrix Formation In Vivo and In Vitro. Tissue Eng. Part B. Rev. 23, 268–280. 10.1089/ten.TEB.2016.0454 27846781 PMC5467150

[B9] Bo S. Gambino R. Ponzo V. Cioffi I. Goitre I. Evangelista A. (2018). Effects of resveratrol on bone health in type 2 diabetic patients. A double-blind randomized-controlled trial. Nutr. Diabetes. 8, 51. 10.1038/s41387-018-0059-4 30237505 PMC6147949

[B10] Burmester G. R. Pope J. E. (2017). Novel treatment strategies in rheumatoid arthritis. Lancet 389, 2338–2348. 10.1016/s0140-6736(17)31491-5 28612748

[B11] Cai X. Chen X. M. Xia X. Bao K. Wang R. R. Peng J. H. (2018). The Bone-Protecting Efficiency of Chinese Medicines Compared With Western Medicines in Rheumatoid Arthritis: A Systematic Review and Meta-Analysis of Comparative Studies. Front. Pharmacol. 9:914:914. 10.3389/fphar.2018.00914 30233362 PMC6134841

[B12] Cai Z. Feng Y. Li C. Yang K. Sun T. Xu L. (2018). Magnoflorine with hyaluronic acid gel promotes subchondral bone regeneration and attenuates cartilage degeneration in early osteoarthritis. Bone 116, 266–278. 10.1016/j.bone.2018.08.015 30149068

[B13] Calderón-Montaño J. M. Burgos-Morón E. Pérez-Guerrero C. López-Lázaro M. (2011). A review on the dietary flavonoid kaempferol. Mini Rev. Med. Chem. 11, 298–344. 10.2174/138955711795305335 21428901

[B14] Cascao R. Vidal B. Jalmari Finnila M. A. Lopes I. P. Teixeira R. L. Saarakkala S. (2017). Effect of celastrol on bone structure and mechanics in arthritic rats. RMD Open 3, e000438. 10.1136/rmdopen-2017-000438 28955491 PMC5604704

[B15] Chan M. A. Kohlmeier J. E. Branden M. Jung M. Benedict S. H. (1999). Triptolide is more effective in preventing T cell proliferation and interferon-gamma production than is FK506. Phytother. Res. 13, 464–467. 10.1002/(sici)1099-1573(199909)13:6<464::aid-ptr483>3.0.co;2-4 10479754

[B16] Chi L. Gao W. Shu X. Lu X. (2014). A natural flavonoid glucoside, icariin, regulates Th17 and alleviates rheumatoid arthritis in a murine model. Mediators Inflamm. 2014:392062. 10.1155/2014/392062 25374443 PMC4211316

[B17] Chun K. H. Jin H. C. Kang K. S. Chang T. S. Hwang G. S. (2020). Poncirin Inhibits Osteoclast Differentiation and Bone Loss through Down-Regulation of NFATc1 In Vitro and In Vivo. Biomol. Ther. (Seoul) 28, 337–343. 10.4062/biomolther.2018.216 31500404 PMC7327144

[B18] Corrado A. Maruotti N. Cantatore F. P. (2017). Osteoblast Role in Rheumatic Diseases. Int. J. Mol. Sci. 18, 1272. 10.3390/ijms18061272 PMC5486094 28617323

[B19] Cui J. Li X. Wang S. Su Y. Chen X. Cao L. (2020). Triptolide prevents bone loss via suppressing osteoclastogenesis through inhibiting PI3K-AKT-NFATc1 pathway. J. Cell Mol. Med. 24, 6149–6161. 10.1111/jcmm.15229 32347017 PMC7294126

[B20] Dejaeger M. Böhm A. M. Dirckx N. Devriese J. Nefyodova E. Cardoen R. (2017). Integrin-Linked Kinase Regulates Bone Formation by Controlling Cytoskeletal Organization and Modulating BMP and Wnt Signaling in Osteoprogenitors. J. Bone Miner. Res. 32, 2087–2102. 10.1002/jbmr.3190 28574598

[B21] Dinesh P. Rasool M. (2018). Berberine inhibits IL-21/IL-21R mediated inflammatory proliferation of fibroblast-like synoviocytes through the attenuation of PI3K/Akt signaling pathway and ameliorates IL-21 mediated osteoclastogenesis. Cytokine 106, 54–66. 10.1016/j.cyto.2018.03.005 29549724

[B22] Ding X. Li W. Chen D. Zhang C. Wang L. Zhang H. (2019). Asperosaponin VI stimulates osteogenic differentiation of rat adipose-derived stem cells. Regener. Ther. 11, 17–24. 10.1016/j.reth.2019.03.007 PMC6518317 31193169

[B23] Doss H. M. Samarpita S. Ganesan R. Rasool M. (2018). Ferulic acid, a dietary polyphenol suppresses osteoclast differentiation and bone erosion via the inhibition of RANKL dependent NF-kappaB signalling pathway. Life Sci. 207, 284–295. 10.1016/j.lfs.2018.06.013 29908722

[B24] Feng Y. L. Jiang X. T. Ma F. F. Han J. Tang X. L. (2018). Resveratrol prevents osteoporosis by upregulating FoxO1 transcriptional activity. Int. J. Mol. Med. 41, 202–212. 10.3892/ijmm.2017.3208 29115382 PMC5746307

[B25] Fujita K. Tokuda H. Kainuma S. Kuroyanagi G. Yamamoto N. Matsushima-Nishiwaki R. (2017). Resveratrol suppresses thyroid hormone−induced osteocalcin synthesis in osteoblasts. Mol. Med. Rep. 16, 2881–2886. 10.3892/mmr.2017.6872 28677796

[B26] Fujiwara T. Zhou J. Ye S. Zhao H. (2016). RNA-binding protein Musashi2 induced by RANKL is critical for osteoclast survival. Cell Death Dis. 7, e2300. 10.1038/cddis.2016.213 27441652 PMC4973353

[B27] Gan K. Xu L. Feng X. Zhang Q. Wang F. Zhang M. (2015). Celastrol attenuates bone erosion in collagen-Induced arthritis mice and inhibits osteoclast differentiation and function in RANKL-induced RAW264.7. Int. Immunopharmacol. 24, 239–246. 10.1016/j.intimp.2014.12.012 25529994

[B28] Gravallese E. M. (2017). Bone Wasn’t Built in a Day: Destruction and Formation of Bone in the Rheumatic Diseases. Trans. Am. Clin. Climatol. Assoc. 128, 24–43. 28790485 PMC5525397

[B29] Gu Y. Fan W. Yin G. (2014). The study of mechanisms of protective effect of Rg1 against arthritis by inhibiting osteoclast differentiation and maturation in CIA mice. Mediators Inflamm. 2014, 305071. 10.1155/2014/305071 25214714 PMC4158307

[B30] Guesmi F. Prasad S. Tyagi A. K. Landoulsi A. (2017). Antinflammatory and anticancer effects of terpenes from oily fractions of Teucruim alopecurus, blocker of IkappaBalpha kinase, through downregulation of NF-kappaB activation, potentiation of apoptosis and suppression of NF-kappaB-regulated gene expression. BioMed. Pharmacother. 95, 1876–1885. 10.1016/j.biopha.2017.09.115 28968948

[B31] Güler-Yüksel M. Hoes J. N. Bultink I. E. M. Lems W. F. (2018). Glucocorticoids, Inflammation and Bone. Calcif. Tissue Int. 102, 592–606. 10.1007/s00223-017-0335-7 29313071

[B32] Guo C. Yang R. J. Jang K. Zhou X. L. Liu Y. Z. (2017). Protective Effects of Pretreatment with Quercetin Against Lipopolysaccharide-Induced Apoptosis and the Inhibition of Osteoblast Differentiation via the MAPK and Wnt/beta-Catenin Pathways in MC3T3-E1 Cells. Cell Physiol. Biochem. 43, 1547–1561. 10.1159/000481978 29035884

[B33] Guo A. J. Choi R. C. Zheng K. Y. Chen V. P. Dong T. T. Wang Z. T. (2012). Kaempferol as a flavonoid induces osteoblastic differentiation via estrogen receptor signaling. Chin. Med. 7:10. 10.1186/1749-8546-7-10 22546174 PMC3350445

[B34] Guo Q. Zheng K. Fan D. Zhao Y. Li L. Bian Y. (2017). Wu-Tou Decoction in Rheumatoid Arthritis: Integrating Network Pharmacology and In Vivo Pharmacological Evaluation. Front. Pharmacol. 8, 230. 10.3389/fphar.2017.00230 28515692 PMC5414545

[B35] Haleagrahara N. Hodgson K. Miranda-Hernandez S. Hughes S. Kulur A. B. Ketheesan N. (2018). Flavonoid quercetin-methotrexate combination inhibits inflammatory mediators and matrix metalloproteinase expression, providing protection to joints in collagen-induced arthritis. Inflammopharmacology 26, 1219–1232. 10.1007/s10787-018-0464-2 29616452

[B36] Halling Linder C. Ek-Rylander B. Krumpel M. Norgård M. Narisawa S. Millán J. L. (2017). Bone Alkaline Phosphatase and Tartrate-Resistant Acid Phosphatase: Potential Co-regulators of Bone Mineralization. Calcif. Tissue Int. 101, 92–101. 10.1007/s00223-017-0259-2 28303318 PMC5486932

[B37] Hao L. Wan Y. Xiao J. Tang Q. Deng H. Chen L. (2017). A study of Sirt1 regulation and the effect of resveratrol on synoviocyte invasion and associated joint destruction in rheumatoid arthritis. Mol. Med. Rep. 16, 5099–5106. 10.3892/mmr.2017.7299 28849139 PMC5647035

[B38] He X. Andersson G. Lindgren U. Li Y. (2010). Resveratrol prevents RANKL-induced osteoclast differentiation of murine osteoclast progenitor RAW 264.7 cells through inhibition of ROS production. Biochem. Biophys. Res. Commun. 401, 356–362. 10.1016/j.bbrc.2010.09.053 20851107

[B39] He L. G. Li X. L. Zeng X. Z. Duan H. Wang S. Lei L. S. (2014). Sinomenine induces apoptosis in RAW 264.7 cell-derived osteoclasts in vitro via caspase-3 activation. Acta Pharmacol. Sin. 35, 203–210. 10.1038/aps.2013.139 24362325 PMC4651217

[B40] He L. Duan H. Li X. Wang S. Zhang Y. Lei L. (2016). Sinomenine down-regulates TLR4/TRAF6 expression and attenuates lipopolysaccharide-induced osteoclastogenesis and osteolysis. Eur. J. Pharmacol. 779, 66–79. 10.1016/j.ejphar.2016.03.014 26965104

[B41] Ho C. T. K. Mok C. C. Cheung T. T. Kwok K. Y. Yip R. M. L. (2019). Management of rheumatoid arthritis: 2019 updated consensus recommendations from the Hong Kong Society of Rheumatology. Clin. Rheumatol. 38, 3331–3350. 10.1007/s10067-019-04761-5 31485846

[B42] Hsieh T. P. Sheu S. Y. Sun J. S. Chen M. H. (2011). Icariin inhibits osteoclast differentiation and bone resorption by suppression of MAPKs/NF-kappaB regulated HIF-1alpha and PGE(2) synthesis. Phytomedicine 18, 176–185. 10.1016/j.phymed.2010.04.003 20554188

[B43] Jia Y. Miao Y. Yue M. Shu M. Wei Z. Dai Y. (2018). Tetrandrine attenuates the bone erosion in collagen-induced arthritis rats by inhibiting osteoclastogenesis via spleen tyrosine kinase. FASEB J. 32, 3398–3410. 10.1096/fj.201701148RR 29401630

[B44] Jia Y. Tao Y. Lv C. Xia Y. Wei Z. Dai Y. (2019). Tetrandrine enhances the ubiquitination and degradation of Syk through an AhR-c-src-c-Cbl pathway and consequently inhibits osteoclastogenesis and bone destruction in arthritis. Cell Death Dis. 10:38. 10.1038/s41419-018-1286-2 30674869 PMC6427010

[B45] Jia Z. Chen A. Wang C. He M. Xu J. Fu H. (2019). Amelioration effects of Kaempferol on immune response following chronic intermittent cold-stress. Res. Vet. Sci. 125, 390–396. 10.1016/j.rvsc.2019.08.012 31412308

[B46] Ke K. Li Q. Yang X. Xie Z. Wang Y. Shi J. (2016). Asperosaponin VI promotes bone marrow stromal cell osteogenic differentiation through the PI3K/AKT signaling pathway in an osteoporosis model. Sci. Rep. 6, 35233. 10.1038/srep35233 27756897 PMC5069473

[B47] Kim B. Lee K. Y. Park B. (2018). Icariin abrogates osteoclast formation through the regulation of the RANKL-mediated TRAF6/NF-kappaB/ERK signaling pathway in Raw264.7 cells. Phytomedicine 51, 181–190. 10.1016/j.phymed.2018.06.020 30466615

[B48] Kim C. J. Shin S. H. Kim B. J. Kim C. H. Kim J. H. Kang H. M. (2018). The Effects of Kaempferol-Inhibited Autophagy on Osteoclast Formation. Int. J. Mol. Sci. 19, 125. 10.3390/ijms19010125 PMC5796074 29301320

[B49] Kim J. H. Kim K. Jin H. M. Song I. Youn B. U. Lee J. (2009). Silibinin inhibits osteoclast differentiation mediated by TNF family members. Mol. Cells 28, 201–207. 10.1007/s10059-009-0123-y 19756392

[B50] Kim J. Y. Cheon Y. H. Yoon K. H. Lee M. S. Oh J. (2014). Parthenolide inhibits osteoclast differentiation and bone resorbing activity by down-regulation of NFATc1 induction and c-Fos stability, during RANKL-mediated osteoclastogenesis. BMB Rep. 47, 451–456. 10.5483/bmbrep.2014.47.8.206 24314143 PMC4206717

[B51] Kim I. R. Kim S. E. Baek H. S. Kim B. J. Kim C. H. Chung I. K. (2016). The role of kaempferol-induced autophagy on differentiation and mineralization of osteoblastic MC3T3-E1 cells. BMC Complement Altern. Med. 16, 333. 10.1186/s12906-016-1320-9 27581091 PMC5007678

[B52] Kim H. R. Kim B. M. Won J. Y. Lee K. A. Ko H. M. Kang Y. S. (2019). Quercetin, a Plant Polyphenol, Has Potential for the Prevention of Bone Destruction in Rheumatoid Arthritis. J. Med. Food. 22, 152–161. 10.1089/jmf.2018.4259 30596535

[B53] Ko J. H. Sethi G. Um J. Y. Shanmugam M. K. Arfuso F. Kumar A. P. (2017). The Role of Resveratrol in Cancer Therapy. Int. J. Mol. Sci. 18, 2589. 10.3390/ijms18122589 PMC5751192 29194365

[B54] Komori T. (2010). Regulation of osteoblast differentiation by Runx2. Adv. Exp. Med. Biol. 658, 43–49. 10.1007/978-1-4419-1050-9_5 19950014

[B55] Komori T. (2019). Regulation of Proliferation, Differentiation and Functions of Osteoblasts by Runx2. Int. J. Mol. Sci. 20, 1694. 10.3390/ijms20071694 PMC6480215 30987410

[B56] Kong X. Zhang Y. Liu C. Guo W. Li X. Su X. (2013). Anti-angiogenic effect of triptolide in rheumatoid arthritis by targeting angiogenic cascade. PloS One 8, e77513. 10.1371/journal.pone.0077513 24204851 PMC3810371

[B57] Kuroyanagi G. Tokuda H. Yamamoto N. Matsushima-Nishiwaki R. Mizutani J. Kozawa O. (2015). Resveratrol amplifies BMP-4-stimulated osteoprotegerin synthesis via p38 MAP kinase in osteoblasts. Mol. Med. Rep. 12, 3849–3854. 10.3892/mmr.2015.3877 26044505

[B58] Law S. K. Simmons M. P. Techen N. Khan I. A. He M. F. Shaw P. C. (2011). Molecular analyses of the Chinese herb Leigongteng (Tripterygium wilfordii Hook.f.). Phytochemistry 72, 21–26. 10.1016/j.phytochem.2010.10.015 21094504

[B59] Lee W. S. Lee E. G. Sung M. S. Yoo W. H. (2014). Kaempferol inhibits IL-1β-stimulated, RANKL-mediated osteoclastogenesis via downregulation of MAPKs, c-Fos, and NFATc1. Inflammation 37, 1221–1230. 10.1007/s10753-014-9849-6 24696323

[B60] Lee C. J. Moon S. J. Jeong J. H. Lee S. Lee M. H. Yoo S. M. (2018). Kaempferol targeting on the fibroblast growth factor receptor 3-ribosomal S6 kinase 2 signaling axis prevents the development of rheumatoid arthritis. Cell Death Dis. 9, 401. 10.1038/s41419-018-0433-0 29540697 PMC5851988

[B61] Li X. J. Jiang Z. Z. Zhang L. Y. (2014). Triptolide: progress on research in pharmacodynamics and toxicology. J. Ethnopharmacol. 155, 67–79. 10.1016/j.jep.2014.06.006 24933225

[B62] Li B. Hu R. Y. Sun L. Luo R. Lu K. H. Tian X. B. (2016). Potential role of andrographolide in the proliferation of osteoblasts mediated by the ERK signaling pathway. BioMed. Pharmacother. 83, 1335–1344. 10.1016/j.biopha.2016.07.033 27571877

[B63] Li J. Li Y. Pan S. Zhang L. He L. Niu Y. (2019). Paeonol attenuates ligation-induced periodontitis in rats by inhibiting osteoclastogenesis via regulating Nrf2/NF-κB/NFATc1 signaling pathway. Biochimie 156, 129–137. 10.1016/j.biochi.2018.09.004 30213522

[B64] Li H. Wang J. Sun Q. Chen G. Sun S. Ma X. (2018). Jatrorrhizine Hydrochloride Suppresses RANKL-Induced Osteoclastogenesis and Protects against Wear Particle-Induced Osteolysis. Int. J. Mol. Sci. 19, 3698. 10.3390/ijms19113698 PMC6275021 30469456

[B65] Li X. He L. Hu Y. Duan H. Li X. Tan S. (2013). Sinomenine suppresses osteoclast formation and Mycobacterium tuberculosis H37Ra-induced bone loss by modulating RANKL signaling pathways. PloS One 8, e74274. 10.1371/journal.pone.0074274 24066131 PMC3774760

[B66] Li Y. Wang S. Wang Y. Zhou C. Chen G. Shen W. (2013). Inhibitory effect of the antimalarial agent artesunate on collagen-induced arthritis in rats through nuclear factor kappa B and mitogen-activated protein kinase signaling pathway. Transl. Res. 161, 89–98. 10.1016/j.trsl.2012.06.001 22749778

[B67] Li Z. Chen C. Zhu X. Li Y. Yu R. Xu W. (2018). Glycyrrhizin Suppresses RANKL-Induced Osteoclastogenesis and Oxidative Stress Through Inhibiting NF-κB and MAPK and Activating AMPK/Nrf2. Calcif. Tissue Int. 103, 324–337. 10.1007/s00223-018-0425-1 29721581

[B68] Lian J. B. (2015). Epigenetic pathways regulating bone homeostasis. Bone 81, 731–732. 10.1016/j.bone.2015.05.036 26036171

[B69] Liu C. Zhang Y. Kong X. Zhu L. Pang J. Xu Y. (2013). Triptolide Prevents Bone Destruction in the Collagen-Induced Arthritis Model of Rheumatoid Arthritis by Targeting RANKL/RANK/OPG Signal Pathway. Evid. Based. Complement Alternat. Med. 2013:626038. 10.1155/2013/626038 23573139 PMC3610373

[B70] Liu W. Qian X. Ji W. Lu Y. Wei G. Wang Y. (2016). Effects and safety of Sinomenine in treatment of rheumatoid arthritis contrast to methotrexate: a systematic review and Meta-analysis. J. Tradit. Chin. Med. 36, 564–577. 10.1016/s0254-6272(16)30075-9 29932627

[B71] Liu W. Zhang Y. Zhu W. Ma C. Ruan J. Long H. (2018). Sinomenine Inhibits the Progression of Rheumatoid Arthritis by Regulating the Secretion of Inflammatory Cytokines and Monocyte/Macrophage Subsets. Front. Immunol. 9:2228:2228. 10.3389/fimmu.2018.02228 30319663 PMC6168735

[B72] Liu K. Liu Y. Xu Y. Nandakumar K. S. Tan H. He C. (2019). Asperosaponin VI protects against bone destructions in collagen induced arthritis by inhibiting osteoclastogenesis. Phytomedicine 63, 153006. 10.1016/j.phymed.2019.153006 31299594

[B73] Lu L. Rao L. Jia H. Chen J. Lu X. Yang G. (2017). Baicalin positively regulates osteoclast function by activating MAPK/Mitf signalling. J. Cell Mol. Med. 21, 1361–1372. 10.1111/jcmm.13066 28158928 PMC5487921

[B74] Luo Y. Liu M. Xia Y. Dai Y. Chou G. Wang Z. (2010). Therapeutic effect of norisoboldine, an alkaloid isolated from Radix Linderae, on collagen-induced arthritis in mice. Phytomedicine 17, 726–731. 10.1016/j.phymed.2010.01.013 20363113

[B75] Ma J. Wang Z. Zhao J. Miao W. Ye T. Chen A. (2018). Resveratrol Attenuates Lipopolysaccharides (LPS)-Induced Inhibition of Osteoblast Differentiation in MC3T3-E1 Cells. Med. Sci. Monit. 24, 2045–2052. 10.12659/msm.905703 29624568 PMC5903312

[B76] Miao C. G. Yang Y. Y. He X. Li X. F. Huang C. Huang Y. (2013). Wnt signaling pathway in rheumatoid arthritis, with special emphasis on the different roles in synovial inflammation and bone remodeling. Cell Signal. 25, 2069–2078. 10.1016/j.cellsig.2013.04.002 23602936

[B77] Miyazono K. Kamiya Y. Morikawa M. (2010). Bone morphogenetic protein receptors and signal transduction. J. Biochem. 147, 35–51. 10.1093/jb/mvp148 19762341

[B78] Nanjundaiah S. M. Venkatesha S. H. Yu H. Tong L. Stains J. P. Moudgil K. D. (2012). Celastrus and its bioactive celastrol protect against bone damage in autoimmune arthritis by modulating osteoimmune cross-talk. J. Biol. Chem. 287, 22216–22226. 10.1074/jbc.M112.356816 22549786 PMC3381183

[B79] Niu Y. Li Y. Huang H. Kong X. Zhang R. Liu L. (2011). a saponin component from Dipsacus asper wall, induces osteoblast differentiation through bone morphogenetic protein-2/p38 and extracellular signal-regulated kinase 1/2 pathway. Phytother. Res. 25, 1700–1706. 10.1002/ptr.3414 21452371

[B80] Ornstrup M. J. Harsløf T. Kjær T. N. Langdahl B. L. Pedersen S. B. (2014). Resveratrol increases bone mineral density and bone alkaline phosphatase in obese men: a randomized placebo-controlled trial. J. Clin. Endocrinol. Metab. 99, 4720–4729. 10.1210/jc.2014-2799 25322274

[B81] Ornstrup M. J. Harsløf T. Sørensen L. Stenkjær L. Langdahl B. L. Pedersen S. B. (2016). Resveratrol Increases Osteoblast Differentiation In Vitro Independently of Inflammation. Calcif. Tissue Int. 99, 155–163. 10.1007/s00223-016-0130-x 27000750

[B82] Oz B. Yildirim A. Yolbas S. Celik Z. B. Etem E. O. Deniz G. (2019). Resveratrol inhibits Src tyrosine kinase, STAT3, and Wnt signaling pathway in collagen induced arthritis model. Biofactors 45, 69–74. 10.1002/biof.1463 30496633

[B83] Pan D. Li N. Liu Y. Xu Q. Liu Q. You Y. (2018). Kaempferol inhibits the migration and invasion of rheumatoid arthritis fibroblast-like synoviocytes by blocking activation of the MAPK pathway. Int. Immunopharmacol. 55, 174–182. 10.1016/j.intimp.2017.12.011 29268189

[B84] Panagopoulos P. K. Lambrou G. I. (2018). Bone erosions in rheumatoid arthritis: recent developments in pathogenesis and therapeutic implications. J. Musculoskelet. Neuronal Interact. 18, 304–319. 30179207 PMC6146189

[B85] Pang J. L. Ricupero D. A. Huang S. Fatma N. Singh D. P. Romero J. R. (2006). Differential activity of kaempferol and quercetin in attenuating tumor necrosis factor receptor family signaling in bone cells. Biochem. Pharmacol. 71, 818–826. 10.1016/j.bcp.2005.12.023 16434028

[B86] Park B. (2014). Triptolide, a diterpene, inhibits osteoclastogenesis, induced by RANKL signaling and human cancer cells. Biochimie 105, 129–136. 10.1016/j.biochi.2014.07.003 25047443

[B87] Perez-Ternero C. Werner C. M. Nickel A. G. Herrera M. D. Motilva M. J. Bohm M. (2017). Ferulic acid, a bioactive component of rice bran, improves oxidative stress and mitochondrial biogenesis and dynamics in mice and in human mononuclear cells. J. Nutr. Biochem. 48, 51–61. 10.1016/j.jnutbio.2017.06.011 28759787

[B88] Qu X. Zhai Z. Liu X. Li H. Ouyang Z. Wu C. (2014). Dioscin inhibits osteoclast differentiation and bone resorption though down-regulating the Akt signaling cascades. Biochem. Biophys. Res. Commun. 443, 658–665. 10.1016/j.bbrc.2013.12.029 24333429

[B89] Rea S. L. Walsh J. P. Layfield R. Ratajczak T. Xu J. (2013). New insights into the role of sequestosome 1/p62 mutant proteins in the pathogenesis of Paget’s disease of bone. Endocr. Rev. 34, 501–524. 10.1210/er.2012-1034 23612225

[B90] Sagar T. Rantlha M. Kruger M. C. Coetzee M. Deepak V. (2016). Ferulic acid impairs osteoclast fusion and exacerbates survival of mature osteoclasts. Cytotechnology 68, 1963–1972. 10.1007/s10616-016-0009-8 27449923 PMC5023575

[B91] Schett G. Emery P. Tanaka Y. Burmester G. Pisetsky D. S. Naredo E. (2016). Tapering biologic and conventional DMARD therapy in rheumatoid arthritis: current evidence and future directions. Ann. Rheum. Dis. 75, 1428–1437. 10.1136/annrheumdis-2016-209201 27261493

[B92] Shen W. Guan Y. Y. Wu R. M. Liu L. X. Li H. D. Bao W. L. (2019). Protective effects of Wang-Bi tablet on bone destruction in collagen-induced arthritis by regulating osteoclast-osteoblast functions. J. Ethnopharmacol. 238:111861. 10.1016/j.jep.2019.111861 30954617

[B93] Singh J. A. Saag K. G. Bridges S. L. Jr. Akl E. A. Bannuru R. R. Sullivan M. C. (2016). 2015 American College of Rheumatology Guideline for the Treatment of Rheumatoid Arthritis. Arthritis Care Res. (Hoboken) 68, 1–25. 10.1002/acr.22783 26545825

[B94] Smolen J. S. Aletaha D. McInnes I. B. (2016). Rheumatoid arthritis. Lancet 388, 2023–2038. 10.1016/s0140-6736(16)30173-8 27156434

[B95] Smolen J. S. Landewe R. Bijlsma J. Burmester G. Chatzidionysiou K. Dougados M. (2017). EULAR recommendations for the management of rheumatoid arthritis with synthetic and biological disease-modifying antirheumatic drugs: 2016 update. Ann. Rheum. Dis. 76, 960–977. 10.1136/annrheumdis-2016-210715 28264816

[B96] Sun S. Wang Y. Zhou Y. (2010). [Research progress on immunosuppressive activity of monomers extracted from Chinese medicine]. Zhongguo Zhong Yao Za Zhi 35, 393–396. 20423014

[B97] Sun Y. Yao Y. Ding C. Z. (2014). A combination of sinomenine and methotrexate reduces joint damage of collagen induced arthritis in rats by modulating osteoclast-related cytokines. Int. Immunopharmacol. 18, 135–141. 10.1016/j.intimp.2013.11.014 24287449

[B98] Tada M. Inui K. Sugioka Y. Mamoto K. Okano T. Koike T. (2016). Reducing glucocorticoid dosage improves serum osteocalcin in patients with rheumatoid arthritis-results from the TOMORROW study. Osteoporos. Int. 27, 729–735. 10.1007/s00198-015-3291-y 26294294

[B99] Verschueren P. C. Lories R. J. Daans M. Théate I. Durez P. Westhovens R. (2009). Detection, identification and in vivo treatment responsiveness of bone morphogenetic protein (BMP)-activated cell populations in the synovium of patients with rheumatoid arthritis. Ann. Rheum. Dis. 68, 117–123. 10.1136/ard.2007.080127 18276742

[B100] Wahab A. Gao K. Jia C. Zhang F. Tian G. Murtaza G. (2017). Significance of Resveratrol in Clinical Management of Chronic Diseases. Molecules 22, 1329. 10.3390/molecules22081329 PMC6152193 28820474

[B101] Wang J. Zhao Q. (2019). Kaempferitrin inhibits proliferation, induces apoptosis, and ameliorates inflammation in human rheumatoid arthritis fibroblast-like synoviocytes. Phytother. Res. 33, 1726–1735. 10.1002/ptr.6364 31155798

[B102] Wang T. Wan D. Shao L. Dai J. Jiang C. (2015). Notoginsenoside R1 stimulates osteogenic function in primary osteoblasts via estrogen receptor signaling. Biochem. Biophys. Res. Commun. 466, 232–239. 10.1016/j.bbrc.2015.09.014 26362186

[B103] Wang J. Qu T. B. Chu L. S. Li L. Ren C. C. Sun S. Q. (2016). [Ligustrazine Promoted the Migration of Bone Marrow Mesenchymal Stem Cells by Up-regulating MMP-2 and MMP-9 Expressions]. Zhongguo Zhong Xi Yi Jie He Za Zhi 36, 718–723. 27491232

[B104] Wang X. He X. Zhang C. F. Guo C. R. Wang C. Z. Yuan C. S. (2017). Anti-arthritic effect of berberine on adjuvant-induced rheumatoid arthritis in rats. BioMed. Pharmacother. 89, 887–893. 10.1016/j.biopha.2017.02.099 28282791

[B105] Wang S. Zuo S. Liu Z. Ji X. Yao Z. Wang X. (2018). Study on the efficacy and mechanism of triptolide on treating TNF transgenic mice with rheumatoid arthritis. BioMed. Pharmacother. 106, 813–820. 10.1016/j.biopha.2018.07.021 29990875

[B106] Wang Q. Ye C. Sun S. Li R. Shi X. Wang S. (2019). Curcumin attenuates collagen-induced rat arthritis via anti-inflammatory and apoptotic effects. Int. Immunopharmacol. 72, 292–300. 10.1016/j.intimp.2019.04.027 31005039

[B107] Wang S. Liu Z. Wang J. Wang Y. Liu J. Ji X. (2019). The triptolide-induced apoptosis of osteoclast precursor by degradation of cIAP2 and treatment of rheumatoid arthritis of TNF-transgenic mice. Phytother. Res. 33, 342–349. 10.1002/ptr.6224 30417444

[B108] Wang Y. Chen H. Zhang H. (2019). Kaempferol promotes proliferation, migration and differentiation of MC3T3-E1 cells via up-regulation of microRNA-101. Artif. Cells Nanomed. Biotechnol. 47, 1050–1056. 10.1080/21691401.2019.1591428 30942633

[B109] Wattel A. Kamel S. Mentaverri R. Lorget F. Prouillet C. Petit J. P. (2003). Potent inhibitory effect of naturally occurring flavonoids quercetin and kaempferol on in vitro osteoclastic bone resorption. Biochem. Pharmacol. 65, 35–42. 10.1016/s0006-2952(02)01445-4 12473376

[B110] Wei Z. F. Jiao X. L. Wang T. Lu Q. Xia Y. F. Wang Z. T. (2013a). Norisoboldine alleviates joint destruction in rats with adjuvant-induced arthritis by reducing RANKL, IL-6, PGE(2), and MMP-13 expression. Acta Pharmacol. Sin. 34, 403–413. 10.1038/aps.2012.187 23396374 PMC4002497

[B111] Wei Z. F. Tong B. Xia Y. F. Lu Q. Chou G. X. Wang Z. T. (2013b). Norisoboldine suppresses osteoclast differentiation through preventing the accumulation of TRAF6-TAK1 complexes and activation of MAPKs/NF-kappaB/c-Fos/NFATc1 Pathways. PloS One 8, e59171. 10.1371/journal.pone.0059171 23536866 PMC3594163

[B112] Wei Z. F. Lv Q. Xia Y. Yue M. F. Shi C. Xia Y. F. (2015). Norisoboldine, an Anti-Arthritis Alkaloid Isolated from Radix Linderae, Attenuates Osteoclast Differentiation and Inflammatory Bone Erosion in an Aryl Hydrocarbon Receptor-Dependent Manner. Int. J. Biol. Sci. 11, 1113–1126. 10.7150/ijbs.12152 26221077 PMC4515821

[B113] Wei C. M. Liu Q. Song F. M. Lin X. X. Su Y. J. Xu J. (2018). Artesunate inhibits RANKL-induced osteoclastogenesis and bone resorption in vitro and prevents LPS-induced bone loss in vivo. J. Cell Physiol. 233, 476–485. 10.1002/jcp.25907 28294321

[B114] Wen L. Zhao Y. Jiang Y. Yu L. Zeng X. Yang J. (2017). Identification of a flavonoid C-glycoside as potent antioxidant. Free Radic. Biol. Med. 110, 92–101. 10.1016/j.freeradbiomed.2017.05.027 28587909

[B115] Xu H. Zhao H. Lu C. Qiu Q. Wang G. Huang J. (2016). Triptolide Inhibits Osteoclast Differentiation and Bone Resorption In Vitro via Enhancing the Production of IL-10 and TGF-beta1 by Regulatory T Cells. Mediators Inflamm. 2016:8048170. 10.1155/2016/8048170 27413257 PMC4930824

[B116] Xu Q. Chen G. Liu X. Dai M. Zhang B. (2019). Icariin inhibits RANKL-induced osteoclastogenesis via modulation of the NF-kappaB and MAPK signaling pathways. Biochem. Biophys. Res. Commun. 508, 902–906. 10.1016/j.bbrc.2018.11.201 30538045

[B117] Yang C. M. Chen Y. W. Chi P. L. Lin C. C. Hsiao L. D. (2017). Resveratrol inhibits BK-induced COX-2 transcription by suppressing acetylation of AP-1 and NF-κB in human rheumatoid arthritis synovial fibroblasts. Biochem. Pharmacol. 132, 77–91. 10.1016/j.bcp.2017.03.003 28288820

[B118] Yang G. Chang C. C. Yang Y. Yuan L. Xu L. Ho C. T. (2018). Resveratrol Alleviates Rheumatoid Arthritis via Reducing ROS and Inflammation, Inhibiting MAPK Signaling Pathways, and Suppressing Angiogenesis. J. Agric. Food Chem. 66, 12953–12960. 10.1021/acs.jafc.8b05047 30511573

[B119] Yoon H. Y. Lee E. G. Lee H. Cho I. J. Choi Y. J. Sung M. S. (2013). Kaempferol inhibits IL-1beta-induced proliferation of rheumatoid arthritis synovial fibroblasts and the production of COX-2, PGE2 and MMPs. Int. J. Mol. Med. 32, 971–977. 10.3892/ijmm.2013.1468 23934131

[B120] Yu Y. Li X. Mi J. Qu L. Yang D. Guo J. (2018). Resveratrol Suppresses Matrix Metalloproteinase-2 Activation Induced by Lipopolysaccharide in Mouse Osteoblasts via Interactions with AMP-Activated Protein Kinase and Suppressor of Cytokine Signaling 1. Molecules 23, 2327. 10.3390/molecules23092327 PMC6225262 30213073

[B121] Yuan X. Tong B. Dou Y. Wu X. Wei Z. Dai Y. (2016). Tetrandrine ameliorates collagen-induced arthritis in mice by restoring the balance between Th17 and Treg cells via the aryl hydrocarbon receptor. Biochem. Pharmacol. 101, 87–99. 10.1016/j.bcp.2015.11.025 26640276

[B122] Yuan Y. Zhang Y. He X. Fan S. (2018). Protective Effects of Sinomenine on CFA-Induced Inflammatory Pain in Rats. Med. Sci. Monit. 24, 2018–2024. 10.12659/msm.906726 29620048 PMC5903310

[B123] Yuan K. Li X. Lu Q. Zhu Q. Jiang H. Wang T. (2019). Application and Mechanisms of Triptolide in the Treatment of Inflammatory Diseases-A Review. Front. Pharmacol. 10:1469:1469. 10.3389/fphar.2019.01469 31866868 PMC6908995

[B124] Zampeli E. Vlachoyiannopoulos P. G. Tzioufas A. G. (2015). Treatment of rheumatoid arthritis: Unraveling the conundrum. J. Autoimmun. 65, 1–18. 10.1016/j.jaut.2015.10.003 26515757

[B125] Zenkov N. K. Chechushkov A. V. Kozhin P. M. Kandalintseva N. V. Martinovich G. G. Menshchikova E. B. (2016). Plant Phenols and Autophagy. Biochem. (Mosc) 81, 297–314. 10.1134/s0006297916040015 27293088

[B126] Zerbini C. A. F. Clark P. Mendez-Sanchez L. Pereira R. M. R. Messina O. D. Una C. R. (2017). Biologic therapies and bone loss in rheumatoid arthritis. Osteoporos. Int. 28, 429–446. 10.1007/s00198-016-3769-2 27796445

[B127] Zhang C. Peng J. Wu S. Jin Y. Xia F. Wang C. (2014). Dioscin promotes osteoblastic proliferation and differentiation via Lrp5 and ER pathway in mouse and human osteoblast-like cell lines. J. BioMed. Sci. 21, 30. 10.1186/1423-0127-21-30 24742230 PMC4014146

[B128] Zhang H. C. Liu M. X. Wang E. P. Lin Z. Lv G. F. Chen X. (2015). Effect of sinomenine on the expression of rheumatoid arthritis fibroblast-like synoviocytes MyD88 and TRAF6. Genet. Mol. Res. 14, 18928–18935. 10.4238/2015.December.28.41 26782542

[B129] Zhang L. Zhu M. Li M. Du Y. Duan S. Huang Y. (2017). Ginsenoside Rg1 attenuates adjuvant-induced arthritis in rats via modulation of PPAR-gamma/NF-kappaB signal pathway. Oncotarget 8, 55384–55393. 10.18632/oncotarget.19526 28903427 PMC5589666

[B130] Zhang Q. Peng W. Wei S. Wei D. Li R. Liu J. (2019). Guizhi-Shaoyao-Zhimu decoction possesses anti-arthritic effects on type II collagen-induced arthritis in rats via suppression of inflammatory reactions, inhibition of invasion & migration and induction of apoptosis in synovial fibroblasts. BioMed. Pharmacother. 118:109367. 10.1016/j.biopha.2019.109367 31545276

[B131] Zhang Y. Wang G. Wang T. Cao W. Zhang L. Chen X. (2019a). Nrf2-Keap1 pathway-mediated effects of resveratrol on oxidative stress and apoptosis in hydrogen peroxide-treated rheumatoid arthritis fibroblast-like synoviocytes. Ann. N. Y. Acad. Sci. 1457, 166–178. 10.1111/nyas.14196 31475364

[B132] Zhang Y. Zou B. Tan Y. Su J. Wang Y. Xu J. (2019b). Sinomenine inhibits osteolysis in breast cancer by reducing IL-8/CXCR1 and c-Fos/NFATc1 signaling. Pharmacol. Res. 142, 140–150. 10.1016/j.phrs.2019.02.015 30797069

[B133] Zhao Y. Li J. Yu K. Liu Y. Chen X. (2007). Sinomenine inhibits maturation of monocyte-derived dendritic cells through blocking activation of NF-kappa B. Int. Immunopharmacol. 7, 637–645. 10.1016/j.intimp.2007.01.007 17386411

[B134] Zhao H. Xu H. Zuo Z. Wang G. Liu M. Guo M. (2018). Yi Shen Juan Bi Pill Ameliorates Bone Loss and Destruction Induced by Arthritis Through Modulating the Balance of Cytokines Released by Different Subpopulations of T Cells. Front. Pharmacol. 9:262:262. 10.3389/fphar.2018.00262 29636683 PMC5880890

[B135] Zhao X. E. Yang Z. Zhang H. Yao G. Liu J. Wei Q. (2018). Resveratrol Promotes Osteogenic Differentiation of Canine Bone Marrow Mesenchymal Stem Cells Through Wnt/Beta-Catenin Signaling Pathway. Cell Reprog. 20, 371–381. 10.1089/cell.2018.0032 31251673

[B136] Zhao Y. Sun X. Yu X. Gao R. Yin L. (2018). Saponins from Panax notoginseng leaves improve the symptoms of aplastic anemia and aberrant immunity in mice. BioMed. Pharmacother. 102, 959–965. 10.1016/j.biopha.2018.03.175 29710551

[B137] Zheng X. Cheng Y. Chen Y. Yue Y. Li Y. Xia S. (2019). Ferulic Acid Improves Depressive-Like Behavior in Prenatally-Stressed Offspring Rats via Anti-Inflammatory Activity and HPA Axis. Int. J. Mol. Sci. 20, 493. 10.3390/ijms20030493 PMC6387299 30678337

[B138] Zhou B. Lu X. Tang Z. Liu D. Zhou Y. Zeng P. (2017). Influence of sinomenine upon mesenchymal stem cells in osteoclastogenesis. BioMed. Pharmacother. 90, 835–841. 10.1016/j.biopha.2017.03.084 28437887

[B139] Zhou F. Shen Y. Liu B. Chen X. Wan L. Peng D. (2017). Gastrodin inhibits osteoclastogenesis via down-regulating the NFATc1 signaling pathway and stimulates osseointegration in vitro. Biochem. Biophys. Res. Commun. 484, 820–826. 10.1016/j.bbrc.2017.01.179 28161640

[B140] Zhu K. J. Shen Q. Y. Cheng H. Mao X. H. Lao L. M. Hao G. L. (2005). Triptolide affects the differentiation, maturation and function of human dendritic cells. Int. Immunopharmacol. 5, 1415–1426. 10.1016/j.intimp.2005.03.020 15953568

[B141] Zhu L. Tang Y. Li X. Y. Keller E. T. Yang J. Cho J. S. (2020a). Osteoclast-mediated bone resorption is controlled by a compensatory network of secreted and membrane-tethered metalloproteinases. Sci. Transl. Med. 12, 6143. 10.1126/scitranslmed.aaw6143 PMC7603424 32024800

[B142] Zhu L. Zhang Z. Xia N. Zhang W. Wei Y. Huang J. (2020b). Anti-arthritic activity of ferulic acid in complete Freund’s adjuvant (CFA)-induced arthritis in rats: JAK2 inhibition. Inflammopharmacology 28, 463–473. 10.1007/s10787-019-00642-0 31562605

[B143] Zhu G. Z. Han X. C. Wang H. Z. Yang Y. Z. Gao Y. Wang H. L. (2019). [Effect of Tripterygium Glycosides Tablets in treating rheumatoid arthritis:a systematic review and Meta-analysis]. Zhongguo Zhong Yao Za Zhi 44, 3358–3364. 10.19540/j.cnki.cjcmm.20190305.004 31602895

[B144] Zhu L. Zhang Z. Xia N. Zhang W. Wei Y. Huang J. (2019). Anti-arthritic activity of ferulic acid in complete Freund’s adjuvant (CFA)-induced arthritis in rats: JAK2 inhibition. Inflammopharmacology 28 (2), 463-473. 10.1007/s10787-019-00642-0 31562605

